# Nanoengineering Approaches Toward Artificial Nose

**DOI:** 10.3389/fchem.2021.629329

**Published:** 2021-02-18

**Authors:** Sanggon Kim, Jacob Brady, Faraj Al-Badani, Sooyoun Yu, Joseph Hart, Sungyong Jung, Thien-Toan Tran, Nosang V. Myung

**Affiliations:** ^1^ Department of Chemical and Environmental Engineering, University of California-Riverside, Riverside, CA, United States; ^2^ Department of Chemical and Biomolecular Engineering, University of Notre Dame, Notre Dame, IN, United States; ^3^ Department of Electrical Engineering, University of Texas at Arlington, Arlington, TX, United States

**Keywords:** artificial nose, optical sensor, electrical sensor array, nanomaterials, electronic nose, optoelectrical sensor, gas sensor

## Abstract

Significant scientific efforts have been made to mimic and potentially supersede the mammalian nose using artificial noses based on arrays of individual cross-sensitive gas sensors over the past couple decades. To this end, thousands of research articles have been published regarding the design of gas sensor arrays to function as artificial noses. Nanoengineered materials possessing high surface area for enhanced reaction kinetics and uniquely tunable optical, electronic, and optoelectronic properties have been extensively used as gas sensing materials in single gas sensors and sensor arrays. Therefore, nanoengineered materials address some of the shortcomings in sensitivity and selectivity inherent in microscale and macroscale materials for chemical sensors. In this article, the fundamental gas sensing mechanisms are briefly reviewed for each material class and sensing modality (electrical, optical, optoelectronic), followed by a survey and review of the various strategies for engineering or functionalizing these nanomaterials to improve their gas sensing selectivity, sensitivity and other measures of gas sensing performance. Specifically, one major focus of this review is on nanoscale materials and nanoengineering approaches for semiconducting metal oxides, transition metal dichalcogenides, carbonaceous nanomaterials, conducting polymers, and others as used in single gas sensors or sensor arrays for electrical sensing modality. Additionally, this review discusses the various nano-enabled techniques and materials of optical gas detection modality, including photonic crystals, surface plasmonic sensing, and nanoscale waveguides. Strategies for improving or tuning the sensitivity and selectivity of materials toward different gases are given priority due to the importance of having cross-sensitivity and selectivity toward various analytes in designing an effective artificial nose. Furthermore, optoelectrical sensing, which has to date not served as a common sensing modality, is also reviewed to highlight potential research directions. We close with some perspective on the future development of artificial noses which utilize optical and electrical sensing modalities, with additional focus on the less researched optoelectronic sensing modality.

## Introduction

**FIGURE 1:**
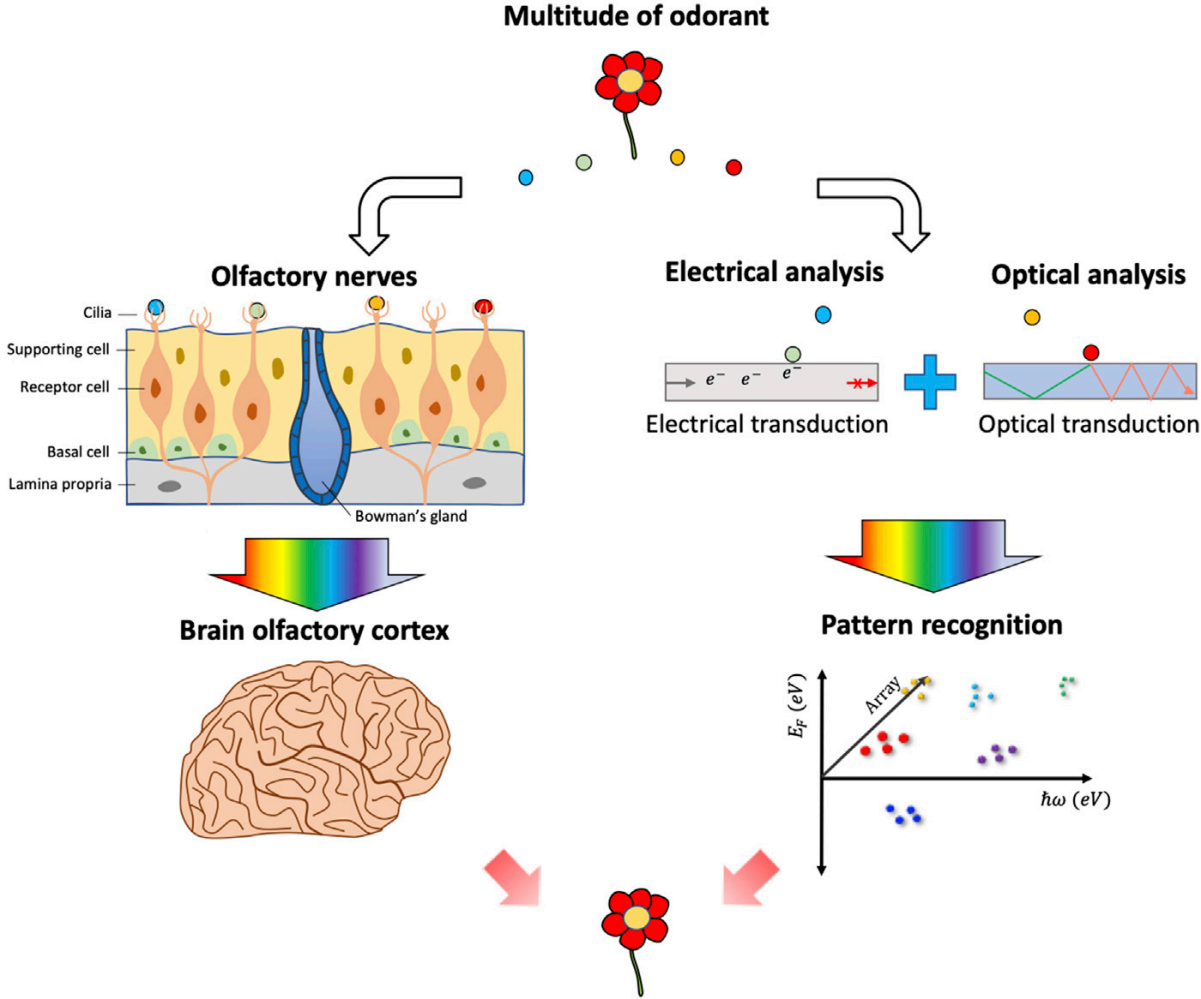
Artificial nose based on electrical and/or optical sensors mimicking the human olfactory system.

The biological olfactory system is highly discriminative and sensitive compared to the other sensory systems. For instance, it has been reported that the human nose can discriminate between 400,000 (Mori and Yoshihara, 1995) up to one trillion different volatile compounds (Bushdid et al., 2014). It is almost impossible to attribute the discriminability of innumerable odors with the “lock and key” theory—that each receptor responds to only one odorant (Mori, 2014)—because humans are known to have only 400 intact olfactory receptors (Niimura, 2012). Additionally, a single odor source typically emits a combination of many unique odorant molecules that vary in composition, rather than a single chemical species (Murthy and Rokni, 2017). This complex task to discriminate and identify odor sources is in part accomplished by the patterns created by all the olfactory receptors, in which a single receptor type can interact with multiple different odorant species (Figure 1) (Buck and Axel, 1991). Therefore, an artificial nose system able to mimic the performance and capabilities of the biological olfactory system is highly desirable for broad applications, especially in areas requiring sensitive chemical detection and odor discrimination.

Thus, for the last two decades, there have been intense efforts to mimic the olfactory systems as the demands for the artificial nose increased for applications that pose potentially serious health and occupational risks for biological noses due to exposure to toxic chemicals, where long-term exposure to the odors may lead to desensitization of the biological nose, and applications requiring quantitative discrimination between very similar odorant mixtures. With the emergence of advanced materials and new sensing mechanisms, the performance of sensors with high specificity of a certain molecule has been improved significantly, leading to the expansion of its application from toxic gases detection to the clinical disease diagnosis via a patient’s breath (Konvalina and Haick, 2014), identification of food contamination or decay, environmental monitoring of pollutants (Kanan et al., 2009a) or explosives (Yang et al., 2015), etc. We refer the reader to (Mahmoudi, 2009; Wilson and Baietto, 2009; Hu et al., 2019b; Karakaya et al., 2020) for additional discussion about the applications of the artificial nose.

Despite considerable and sustained efforts to mimic the biological olfactory system, it remains challenging to detect and discriminate odor molecules in complex gaseous samples where the many odorant species coexist. Artificial nose systems that employ sensor array-based strategies have demonstrated the inherent ability to incorporate a variety of unique sensing materials to generate distinct response patterns to chemical analytes. Array-based strategies implement a series of semi-selective chemical sensors and pattern recognition methods to distinguish and quantify gaseous analytes in complex mixtures, emulating the biological olfactory systems (Gardner and Bartlett, 1994; Capelli et al., 2014; Crespo et al., 2019). Nanoengineered materials allow us to pack multiple sensor arrays on a limited footprint so that they are seen as an essential element to mimic the artificial nose. Also, the reduction of the sensing volume increases the number of atoms on the surface, providing larger interaction sites with the odorants. Besides the small spatial footprint and large surface-area-to-volume ratio, a unique advantage of nanostructured materials is that their size and structure can be precisely controlled to tune their electrical and optical properties. Tunability of both properties by size and structure can confer an additional ability to modulate the electron-matter or photon-matter interaction within a single sensing element, allowing for the generation of more diverse response patterns toward odorants (Figure 1). For example, in Au nanorod, its asymmetric structure allows for the selective excitation of transverse and longitudinal surface plasmon modes by adjusting the excitation wavelength and polarization (Chang et al., 2010). Subsequently, electric field distribution, even within a single nanostructure, can be modulated. In this respect, this review paper covers the recent exceptional achievement in gas sensors by facilitating the quantum effects of nanoengineered materials with special emphasis on the strategies for achieving diverse sensor response patterns to facilitate algorithmic identification and quantification of chemical analytes in artificial nose system. Furthermore, we highlight novel attempts that combine electrical and optical transductions to generate more diverse response patterns for enhanced odorant discrimination.

## Types of Artificial Noses Based on Nanoengineered Materials

### Electrical Approach

Development of sensor array technologies based on electrical detection principles has been rapidly growing owing to several key performance benefits, which include low cost, fast response, reactivity to a broad range of target gases, and ease of miniaturization of sensor and readout electronics. Sensors based on electrical detection transduce chemical interactions to various electrical responses depending on the electrical properties of the sensing materials, the sensor architecture, and the corresponding electrical measurement technique. Sensors employing field-effect transistor (FET) configuration modulate the flow of charge carriers in the semiconducting sensing material bridging the source and drain electrodes via external voltage (i.e., electric field) applied between the gate and source electrodes (Hu et al., 2019b). Upon interaction with the sensor and in combination with the operator-controlled gate voltage and source-drain bias voltage, the gas molecules further modulate the electrical FET characteristics of the device such as the charge carrier mobility, threshold voltage, and subthreshold swing, which, in addition to electrical resistance or conductivity, are multiparametric quantitative sensing features that further contribute to generation of unique sensor response patterns for enhancing the sensor system’s ability to discriminate odorant molecules (Wang et al., 2016). Compared to the three-terminal configuration of FET-based sensors, chemiresistive (or chemoresistive) sensors omit the use of the gate electrode to exert the external electric field, and solely rely on the modulation of device electrical characteristics (i.e., resistance or conductance) through chemical interactions between the gas molecules and sensing channel (Tran and Mulchandani, 2016). Thus, FET-based chemical detection inherently provides more quantitative sensor response features than chemiresistor-based detection methods, but this comes at the cost of more complex device architecture, electrical measurement techniques, and readout instrumentation.

To realize electrical sensor arrays possessing semi-selective binding to a broad range of VOCs that can produce the diverse response patterns needed either for specific sensing applications or for artificial nose applications, diverse and extensive libraries of sensing materials have been investigated to fabricate the sensors and sensor arrays. Sensing materials for chemiresistive and FET-based artificial nose applications have semiconducting properties, which can further be categorized based on composition, such as metal oxide semiconductors (MOS), conducting polymers, carbon nanomaterials-based semiconductors (*e.g.,* single-walled carbon nanotubes, aka. SWNTs), and more recently, transition metal dichalcogenides (TMDCs) (Kim et al., 2014; Cho et al., 2017; Shao et al., 2018; Sun et al., 2019a; Kumar et al., 2020a), and even hybrid nanostructures of these semiconducting materials (Barisci et al., 2002; Oliveros et al., 2002; Berna, 2010; Shirsat et al., 2012; Liu et al., 2015c; Ji et al., 2019; Luna et al., 2019; Torad and Ayad, 2019; Su et al., 2020). Additionally, by controlling morphologies, nanostructures, and even heterostructures of sensing materials, especially at the nanoscale, chemical sensing mechanisms and sensing properties (*i.e.,* sensitivity and selectivity) of these materials can further be modulated to introduce new physical phenomena, such as enhanced photochemical activity, hot carrier injection, and photothermal effect induced through local surface plasmon resonance (Park et al., 2009; Ab Kadir et al., 2014; Luna et al., 2019). Furthermore, the different chemical, physical, and material properties of these sensing materials have been exploited to enable various chemical functionalization/modification strategies to systematically impart secondary chemical and physical behaviors (Blondeau, 2015; Schroeder et al., 2019b). For example, integration of photoactive materials (e.g., metal nanoparticles, macrocyclic dye molecules, etc.) with sensing materials, such as metal oxide semiconductors (MOS) and single-walled carbon nanotubes (SWNTs), allowed for photoelectrochemical modulation of the nanostructured hybrid sensing material’s gas sensing properties (*i.e.,* selectivity and sensitivity) toward analytes (Cho and Park, 2016; Su et al., 2020).

Strategies for nanoengineering of sensing materials (and device architecture, measurement techniques, etc.) can be attributed to tune or enhance one or more specific sensor performance characteristics such as signal-to-noise ratio, limit of detection (for specific analytes), selectivity to certain types of analytes, and stability. Selectivity and sensitivity of individual sensors play crucial roles in contributing to the artificial nose’s ability to qualitatively discriminate and quantitatively measure odorant concentrations, respectively (Gardner and Bartlett, 1994). The receptor function of a sensing material provides the variable selectivity to the individual sensor element.

#### Semiconducting Nanomaterials in Electrical Gas Sensor Arrays

##### Metal Oxide Semiconductors

Electronic noses and sensor arrays based on metal oxides semiconductors have recently been used in food quality analysis (Konduru et al., 2015a; Konduru et al., 2015b; Mei et al., 2015; Xu et al., 2017; Ghasemi-Varnamkhasti et al., 2018a, Ghasemi-Varnamkhasti et al., 2018b; Liu et al., 2018; Tohidi et al., 2018; Ghasemi-Varnamkhasti et al., 2019; Voss et al., 2019a; Voss et al., 2019b; Gamboa et al., 2019; Grassi et al., 2019; Hidayat et al., 2019; Jong et al., 2019; Liang et al., 2019; Zappa, 2019; Lee et al., 2020a; Mu et al., 2020; Rodriguez-Mendez and de Saja, 2020; Tang et al., 2020), medical diagnostics (Moon et al., 2018; Kononov et al., 2019; Liao et al., 2020; Tiele et al., 2020), environmental monitoring (Plawiak and Rzecki, 2014; Yi et al., 2014; Mondal et al., 2015; Akamatsu et al., 2017; Krivetskiy et al., 2017; Pan et al., 2017; Luo et al., 2018; Shi et al., 2018; Gancarz et al., 2019; Jaeschke et al., 2019; Lagod et al., 2019; Liu et al., 2019a; Palacín et al., 2019; Song et al., 2019; Kang et al., 2020; Khan et al., 2020; Prajapati et al., 2020; Rahman et al., 2020; Wu et al., 2020; Yang et al., 2020) and drug detection (Hernandez et al., 2020) applications, among others. While single metal oxide sensors are typically limited by poor selectivity, their incorporation into sensor arrays results in an improved ability to differentiate between single VOCs and VOC mixtures due to the unique response characteristics of different MOS sensing materials (Prajapati et al., 2020). Further selectivity optimization has been achieved through applying temperature modulation (Nakhleh et al., 2017; Prajapati et al., 2020) to the sensors, as MOS gas sensitivity is functional to temperature. Although some reports have discussed room temperature sensing mechanisms (Li et al., 2017; Abokifa et al., 2018; Li et al., 2019; Bindra and Hazra, 2019; Zhang et al., 2020a), MOS sensors typically require higher operating temperatures and thus the incorporation of a heater into the device architecture for operation. Additionally, MOS sensors tend to have nonlinear responses (Bochenkov and Sergeev, 2010), but highly linear results were obtained by applying dielectric excitation to an MOS sensor array recently (Potyrailo et al., 2020).

The sensing mechanism of MOS materials depends on temperature and the specific metal oxide material. Above 700°C, bulk defect effects dominate the conductivity response to changes in the partial pressure of oxygen, which achieves an equilibrium with the stoichiometric bulk material (Bochenkov and Sergeev, 2010). Surface conductance effects are most evident in the 400–600°C range and depend on the adsorption/desorption of monatomic oxygen (Bochenkov and Sergeev, 2010; Reghu et al., 2018). The active metal oxide material forms a sensing film which is designed to optimize mass transfer properties with both oxygen and analyte gases (Bochenkov and Sergeev, 2010), which has been accomplished by increasing the aspect ratio of these materials by depositing layers of hollow, porous, nanospherical active metal oxide material (Kanan et al., 2009b; Righettoni et al., 2015). MOS materials are polycrystalline in nature, with crystallographic faces functioning as the active sites for oxygen adsorption (Bochenkov and Sergeev, 2010) separated by grain boundaries (Korotcenkov, 2007; Reghu et al., 2018). Adsorbed oxygen attracts electrons from the bulk material, creating a space-charge layer which repels other electrons from the surface, thus forming an “electron depletion” layer. With fewer charge carriers (electrons) in the material, conductivity decreases and a potential barrier forms at the grain boundaries (Kanan et al., 2009b; Bochenkov and Sergeev, 2010). In the case of n-type metal oxides, exposure to reducing (electron-donating) gases and their subsequent adsorption to and reactions at the material surface results in more available charge carriers in the conduction band, resulting in reduction of the potential barrier at grain boundaries (Kanan et al., 2009b) and increased conductivity, with the inverse electrical effect observed with oxidizing gases. (Korotcenkov, 2007; Bochenkov and Sergeev, 2010; Righettoni et al., 2015; Reghu et al., 2018).

One strategy for tuning the gas sensing properties of nanoscale metal oxides is to selectively promote high-index crystallographic facets on the surface of the nanostructures which expose additional catalytically favorable active sites for oxygen adsorption and surface reactions with the target analytes, chiefly unsaturated metal ions with a large dangling bond density. The careful selection and control of synthesis conditions are important for promoting growth of the high-energy high index facets over low-energy, less-active low index facets. The interested reader is directed to a review which discusses synthesis strategies of and other information about high-index faceted metal oxides (Sun et al., 2019b) and a review which provides an overview of facet-engineered semiconducting metal oxides in gas sensors (Gao and Zhang, 2018). The engineering of surface facets and morphology for enhanced selectivity and sensitivity has been previously reported for WO_3_ (Hu et al., 2020), TiO_2_ (Liu et al., 2017; Wang et al., 2017), CuO (Hou et al., 2018) and SnO_2_ (Liu et al., 2015a; Zhang et al., 2020c).

Beyond facet engineering, doping nanostructured metal oxides with homogenous, substitutional additives and heterogeneous nanostructures to modify the surface chemical reactivity and electrical properties to enhance sensitivity, selectivity and other gas sensing properties has been the subject of a significant body of research and publications. Only a small selection of works to demonstrate the previous progress or potential to use such techniques in electronic noses is discussed here; the reader is directed to the reviews by (Degler et al., 2019) and (Zhang et al., 2020b) for detailed reviews of homogeneous and heterogeneous metal oxide nanostructures for gas sensing, including the mechanisms by which dopants modify the chemical and electronic properties of metal oxides to improve sensitivity and selectivity.

Absent photoexcitation, noble and transition metal nanoparticle doping has been used as a successful strategy to improve the sensitivity and selectivity of metal oxides by creating Schottky barriers to increase electron-hole recombination time and modifying catalytic activity at the surface (Zhang et al., 2020b). *Liu et al.* demonstrated that decorating the surface of high-index {211}-faceted SnO_2_ with Au nanoparticles improved the selectivity of the hybrid material toward ethanol over acetone, THF and methanol, and increased the sensitivity toward all analytes, albeit barely for methanol. (Liu et al., 2015a) The undecorated SnO_2_ had similar responses toward acetone and ethanol, while Pd and Pt doping significantly reduced the sensitivity toward all analytes. However, despite the lowered sensitivity, Pt imparted somewhat greater selectivity toward methanol. In other works, a combination of doping Cr and/or Pt into a SnO_2_ thin film with temperature modulation allowed for selectivity toward NO_2_ and CO to be attained separately, (Kamble and Umarji, 2016) and greater selectivity toward triethylamine over several other VOCs was achieved by the addition of Pd nanoparticles (present as PdO) onto ZnO nanorods. (Meng et al., 2019) Tangirala et al. (Tangirala et al., 2017) investigated the effects of Cu-, Pt- and Pd doping in nanostructured SnO_2_ powders, and determined that higher sensitivity toward CO was obtained using Cu nanoparticles as a dopant while also noting the impact that two different methods of introducing the dopants had on morphology of the SnO_2_ structures and, consequently, the observed gas sensing performance. In summary, by imparting different catalytic activities and sensitivities toward different VOCs, metal nanoparticle doping is a potentially useful strategy to construct cross-sensitive and semi-selective metal oxide sensor arrays.

A virtual sensor array may also be constructed using a few, or even single, metal oxide sensing materials cycling through different operating temperatures. Metal oxides display an optimum operating temperature with respect to maximizing their response (or sensitivity) toward a specific analyte at a specific concentration due to the strong temperature dependence of and competition between oxygen adsorption, analyte adsorption and surface reaction kinetics (Ahlers et al., 2005). Thus, operating a metal oxide sensor at different temperatures is a viable strategy to distinguish between different analytes that may otherwise be difficult to distinguish at a single operating temperature, provided that the relationships between temperature and sensitivity for the target analytes and the metal oxide sensing material are sufficiently different. Several examples of such virtual sensor arrays and electronic noses using single or a few different metal oxide sensing materials with transient temperature cycling or variation in their operation have been previously reported (Martinelli et al., 2012; Amini et al., 2013; Fonollosa et al., 2013; Warmer et al., 2015; Hastir et al., 2016; Kneer et al., 2016; Krivetskiy et al., 2017; Durán et al., 2018; Krivetskiy et al., 2018; Tonezzer et al., 2018; Tonezzer et al., 2019a; Tonezzer et al., 2019b; Liu et al., 2019b).

##### Graphene and Carbon Nanotubes

Low-dimensionality carbon nanomaterials such as carbon nanotubes (CNTs) and graphene have demonstrated potential for applications in chemical sensor development, especially for artificial nose applications (Park et al., 2010; Wongchoosuk et al., 2010; Son et al., 2016; Orzechowska et al., 2019; Hayasaka et al., 2020). These carbon allotropes exhibit excellent carrier mobility and low thermal and electric noises owing to their bond structure, which is rich in sp^2^ electrons. In addition, these carbon nanomaterials display high mechanical strength and thermal conductivity. Due to the conductive properties and optical transparency, these materials make great candidates for transparent devices for sensor applications (Yusof et al., 2019).

CNTs and graphene are composed of sp^2^ bonded carbon atoms packed into honeycomb lattice structures (Varghese et al., 2015; Tran and Mulchandani, 2016; Shi et al., 2018); graphene has a planar (2-D) atomic monolayer structure, while CNTs assume a cylindrical pseudo 1-D structure resembling a seamlessly rolled sheet of graphene along the (m,n) lattice vector in the sheet (Aroutiounian, 2015; Liu et al., 2015c; Yusof et al., 2019). Divided into two categories based on the number of concentric atomic layers, single-walled carbon nanotubes (SWNTs) can exhibit either semiconducting or metallic electronic properties depending on chirality while and multi-walled carbon nanotubes (MWNTs) have metallic electronic properties (Gong et al., 2005; Tran and Mulchandani, 2016). Semiconducting SWNTs typically have small band gaps of 0.1–0.2 eV, which allow for room-temperature operation (Wongchoosuk et al., 2010; Aroutiounian, 2015; Zaporotskova et al., 2016). This suggests low-power requirement, which is an attractive performance feature for chemical sensors, especially in artificial nose applications that employ high-density sensor arrays. On the other hand, pristine graphene exhibit ambipolar electric field effect, which can operate in both n-type and p-type regimes (Novoselov, 2004; Geim and Novoselov, 2007; Castro Neto et al., 2009).

The chemical sensing mechanism of chemiresistive/FET-based SWNTs sensors can be categorized by intra-tube, inter-tube, and tube-electrode conduction pathways (Schroeder et al., 2019b). Intra-tube chemical sensing mechanisms are governed by modulations in charge carrier concentrations and mobility and which can occur through charge transfer, charge carrier trapping, charge scattering, and any of perturbations of ideal SWNTs structure by chemical and electrostatic interactions on the walls of SWNTs. Inter-tube conduction pathways in sensors based on a network of CNTs can also be modulated by small physical changes in tube-tube junction distance due to intercalation of analytes in the interstitial spaces, influencing the charge tunneling probability in the CNT network. Another sensing mechanism occurs through the modulation of the Schottky barrier at the tube-electrode junction (Heller et al., 2008; Salehi-Khojin et al., 2011; Lerner et al., 2012; Orzechowska et al., 2019; Schroeder et al., 2019b). Similar to SWNTs-based devices, graphene-based chemiresitive and FET-based sensors operate via charge transfer phenomena that occur between the sensing material and adsorbed gas analytes, where charge transfer direction and quantities depend on the electron-donating or electron-withdrawing nature of the analyte molecule (Yang et al., 2017; Donarelli and Ottaviano, 2018).

For chemiresistive and FET-based gas sensing applications with implications toward development of electronic nose system, semiconducting SWNTs and graphene have been extensively utilized as sensing materials, which was reviewed in various references (Dai et al., 2002; Zhang et al., 2008; Liu et al., 2012; Llobet, 2013; Varghese et al., 2015; Zaporotskova et al., 2016; Tang et al., 2017; Xu et al., 2018b; Orzechowska et al., 2019). Specifically for artificial nose applications, efforts to tune the selectivity and sensitivity of SWNT- and graphene-based chemiresistive and FET sensors take advantage of the rich library of covalent and non-covalent chemical modification strategies and the ease of integration or hybridization with a plethora of other chemicals and nanomaterials to impart additional receptor functionalities. Thus, the lack of selectivity of these graphitic nanomaterials have been overcome by introducing secondary sensing materials of various chemical composition and structures to the carbon structure to provide additional receptor functionality, ranging from macrocyclic molecules (e.g., porphyrins, phthalocyanines, pyrenes) (Su et al., 2020) DNA polymers, organic polymers), nanoparticles of various composition (e.g., metallic and semiconducting) (Mubeen et al., 2007; Mubeen et al., 2010; Mubeen et al., 2011), to larger peptide-based receptors (Kuang et al., 2010; Son et al., 2016).

One example of array-based artificial nose using carbon nanomaterials was demonstrated by Schroeder et al. using a 20-sensor array based on SWNTs noncovalently functionalized with 20 different “chemical selector” as sensing materials for identification of various food item categories (Schroeder et al., 2019a). These 20 chemical selectors, used to impart chemical selectivity to bare SWNTs, consisted of transition metal complexes, ionic liquids, porous polymers, cavitands molecules, and metalloporphyrins, which were selected based on their differential affinities to various classes of odorant molecules (Lin and Swager, 2018; Schroeder et al., 2019a). By combining machine learning approaches to guide the experimental design and characterization of the chemical sensor array, *Schroeder et al.* demonstrated the ability to differentiate between various samples of cheese, liquor, and oils by their odors (Schroeder et al., 2019a).

Due to their unique chemical structures, porphyrins have the ability to bind with different analytes through van der Waal forces, hydrogen bonding, electrostatic and coordination interactions with the central metal ion (Brunink et al., 1996). The interaction of porphyrins and their complexes with metals affects the delocalization of the electron charge in graphene and nanotubes, as well as the energy barrier and the size of energy gaps between valence and conductivity bands. In consequence, the conductivity in graphene can change significantly. Based on other experimental and theoretical studies, it was observed that the sensitivity of graphene-based sensors can be significantly improved by doping with Br, N, P, Ga, Cr, Mg, S, and Si (Orzechowska et al., 2019). By utilizing doping, new active sites are formed on the graphene’s surface, which have an ability to strongly adsorb gas molecules (Orzechowska et al., 2019). For example, doping graphene with Fe, N, and N and Si combined improves sensitivity toward H_2_S, CO, and NO_2_ (Zhang et al., 2014) while doping graphene with Mg and Cr results in an increase in sensitivity toward SO_2_ (Shao et al., 2013; Liu et al., 2014; Orzechowska et al., 2019). The nitrogen atom is the active site of NO_2_ adsorption in the N and Si doping, whereas doping graphene with Si significantly improves the sensitivity toward NO and NO_2_. The sensor built of N-Si-G shows sensitivity toward 21 ppm NO_2_ and the sensor response declines along with the gas concentration to ∼1 ppm (Niu et al., 2013). In addition, the introduction of defects in the graphene structure by doping with Br, S and N results in improved sensitivity toward formaldehyde (Zhou et al., 2014).

Another emerging strategy for achieving an artificial nose system is the integration of protein-based olfactory receptors (ORs) into the sensing materials which have been previously demonstrated by several groups to detect VOCs in gas phase and liquid phase down to the ppt level using CNT-based and graphene-based FET sensors (Goldsmith et al., 2011; Lee et al., 2012b; Son and Park, 2018). This biomimetic approach uses the ORs, which are protein-based receptors with multiple genotypic variants found in biological olfaction (over 350 different found in humans), to confer differential affinity of sensors to various gaseous odorants, depending on specific variants used (Lee et al., 2012b). The sensing mechanism using these OR-functionalized sensing materials was proposed to be attributed to the change in local ionization of specific amino acid residues in the OR upon binding to odorants, leading to local electrical modulation of the semiconducting sensing material (Lee et al., 2012b). This approach to integrate ORs onto transduction and sensing materials harnesses the natural selectivity of these polypeptide macromolecules to enable discrimination between odorants by electrical sensors and sensor arrays (Son and Park, 2018).

##### 2D Transition Metal Dichalcogenides

2D semiconducting *transition metal dichalcogenide* (TMDC) nanomaterials, which are binary compounds with the general formula MX_2_ — M being a transition metal and X being a group VI element other than oxygen (i.e., S, Se, Te) — have been extensively engineered and evaluated for their gas sensing performance in FET and chemiresistive measurement configurations. Their use in multimodal sensor arrays and electronic noses is currently not as widespread compared to other materials, but they nonetheless present as promising candidates for the low to moderate temperature sensing of various analytes and incorporation into electronic noses. The few applications of TMDC-based chemiresistive sensor arrays reported in the literature include discriminating between atmospheric pollutants using Zn^2+^-doped MoS_2_ nanosheets of variable composition (Shao et al., 2018), precursors to the explosive triacetone peroxide using variable-composition reduced graphene oxide/MoS_2_ nanocomposites (Sun et al., 2019a) and oxidized/non-oxidized VOCs using organic ligand-functionalized and Au nanoparticle-doped MoS_2_ (Kim et al., 2014; Cho et al., 2017). *Kumar et al.* (Kumar et al., 2020a) recently reviewed 2D nanostructured TMDCs as gas sensors on flexible substrates, with a focus on MoS_2_, MoSe_2_, WS_2_, WSe_2_, SnS_2_ (which is technically not a TMDC but has similar properties and applications to gas sensing), TaS_2_ and VS_2_-based sensors. Many of the sensors they reviewed operated at ambient temperature and displayed higher selectivity toward NO_2_, NH_3_ or humidity. Additionally, 2D NbS_2_ (Kim et al., 2019) and ReS_2_ (Martín-García et al., 2019, 2) nanostructures have recently been demonstrated to act as sensitive room-temperature chemiresistors toward NO_2_ and NH_3_/VOCs, respectively. MoTe_2_ chemiresistors and FETs have also been demonstrated to respond to NH_3_ and NO_2_ (Feng et al., 2017a; Shackery et al., 2018; Rani et al., 2019), with higher sensitivity and improved limits of detection under UV light excitation (Feng et al., 2017b; Wu et al., 2018b). These examples indicate that surface functionalization, doping and photoexcitation are among the tools that can be used to tune the selectivity of TMDC gas sensors and enable their incorporation into electronic nose sensor arrays.

TMDCs display a wide range of electrical properties with metallic, semiconducting and insulating behavior represented by different materials. Group VI TMDCs are narrow-bandgap semiconductors (approximately 1–2 eV optical bandgaps) whose electrical, optical and optoelectrical properties have enabled their use in electrical and optical sensors. The reader is referred to the review by Liu et al. (2015b) for a detailed review of the electronic structure and properties of molybdenum and tungsten 2D TMDCs, which are common TMDCs for gas sensing applications.

Top-down and bottom-up procedures have been employed to synthesize 2D TMDC nanomaterials for use in various gas sensors. *Samadi et al.* provide a detailed review of the various top-down and bottom-up synthesis methods used to make TMDC nanomaterials (Samadi et al., 2018). Of the various synthesis methods, liquid exfoliation is particularly suited for sensor array fabrication in an electronic nose. The result of liquid exfoliation is a suspension of nanosheets which is readily amenable to dropcasting or inkjet printing, both of which are applicable to the mass-fabrication of microelectronics. Typically, the suspension of nanosheets resulting from liquid exfoliation cannot be immediately inkjet printed, as its viscosity and surface tension must be modified with additives to make it printable with an inkjet printer. In a bid to simplify this process, *Lee et al.* recently demonstrated the inkjet printing of MoS_2_, WS_2_, MoSe_2_ and WSe_2_ nanosheets for optical sensor arrays using a simple aqueous exfoliation process with alkyl zwitterion additives; no organic solvents or other additives were required to adjust the fluid properties to make the suspensions inkjet-printable (Lee et al., 2020b). The interested reader is directed to two books about 2D TMDCs for more detailed information about their various properties and other applications (Kolobov and Tominaga, 2016; Arul and Nithya, 2020).

**FIGURE 2:**
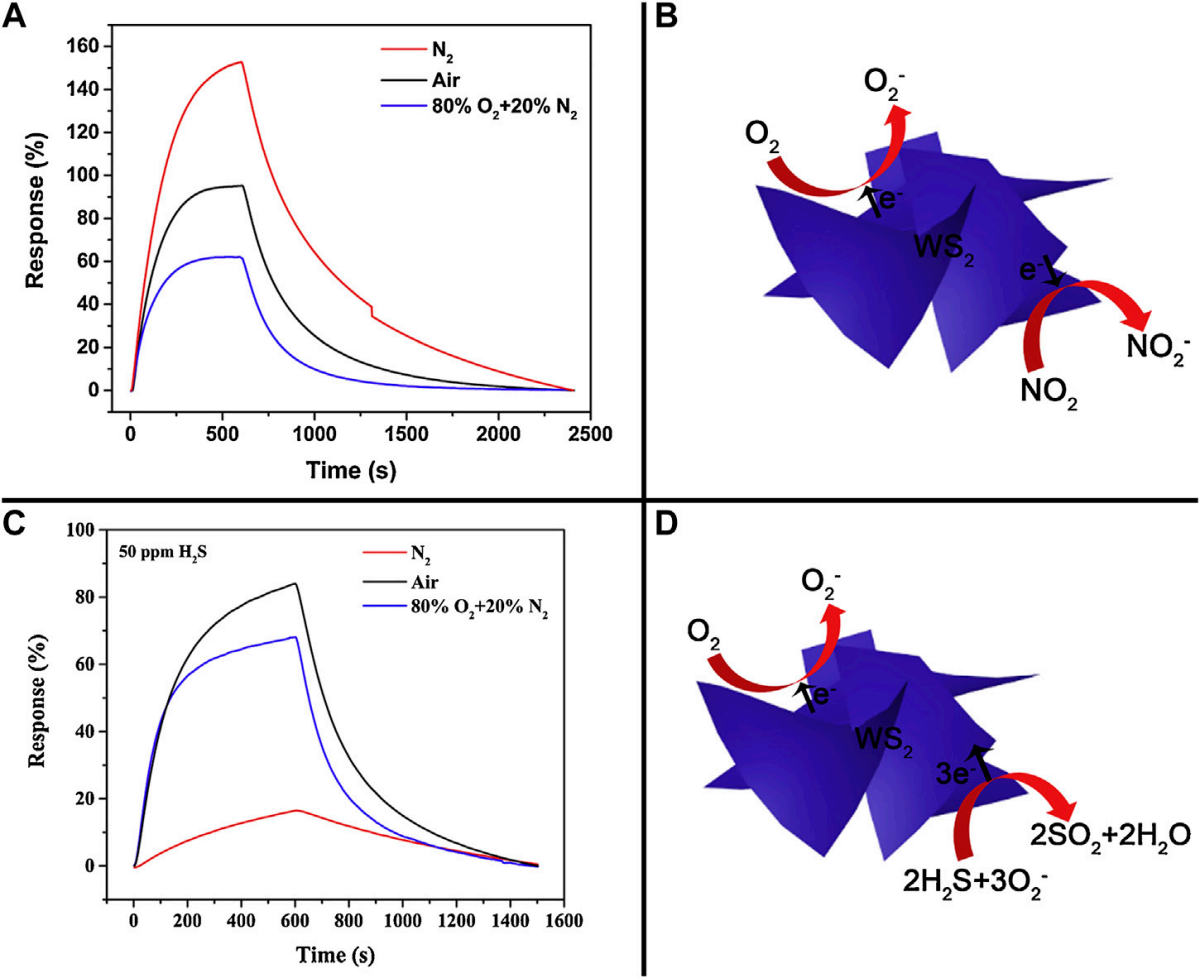
The adsorption of O_2_ onto WS_2_ **(B)** competes with the adsorption of NO_2_, resulting in a decreased response toward NO_2_ at higher O_2_ concentrations **(A)**. H_2_S undergoes weak physisorption and charge transfer in the absence of oxygen, but a redox reaction when adsorbed O_2_ is present **(D)** increases the sensitivity toward H_2_S, displaying an optimum oxygen concentration with respect to sensitivity **(C)**. The sensor was operated at 160°C for the measurements in **(A)** and **(C)**. Reprinted from Sensors and Actuators B: Chemical, 303, Liu et. al, Comparative study on NO_2_ and H_2_S sensing mechanisms of gas sensors based on WS_2_ nanosheets, 127114, Copyright 2019, with permission from Elsevier. (Liu et al., 2020).

Similar to graphene, the gas sensing mechanism of TMDCs typically results from the reversible adsorption of an analyte followed by a charge-transfer process between the analyte and TMDC, which changes the charge carrier concentration in the TDMC, resulting in the conductivity of the material changing. Such a mechanism has been posited for the interaction between SnS_2_ and NO_2_ (Ou et al., 2015), WS_2_ and NO_2_ (Liu et al., 2020), MoSe_2_ and NO_2_ (Chen et al., 2019b), MoSe_2_ and NH_3_ (Late et al., 2014), and MoS_2_ and various gases (Yue et al., 2013). This simple model of analyte adsorption and charge transfer is not applicable in all cases, however. *Liu et al.* demonstrated that an optimal concentration of absorbed oxygen mediates and enhances the response of WS_2_ to H_2_S likely via a surface redox reaction producing SO_2_, similar to the mechanism of semiconducting metal oxide gas sensing, while the absorbed oxygen competes with the physisorption of NO_2_ onto the surface (Figure 2). (Liu et al., 2020) Similarly, the competition between the physisorption/charge transfer and redox/direct electron transfer mechanisms of VOC gas sensing remains a matter of uncertainty with MoS_2_ (Chen et al., 2019a). Thus, the sensing mechanism of TMDCs toward certain analytes in the presence of oxygen may shift to a redox/direct electron transfer-driven process compared to when it is absent and only physisorption/charge transfer can occur, a factor that should be carefully considered in the design and evaluation of TMDC-based gas sensors for a specific application. Physisorption dominates in an inert atmosphere, such as pure nitrogen, while the introduction of oxygen may enable a redox pathway for the oxidation of VOCs at the surface of and subsequent electron release into the TMDC material.

**FIGURE 3:**
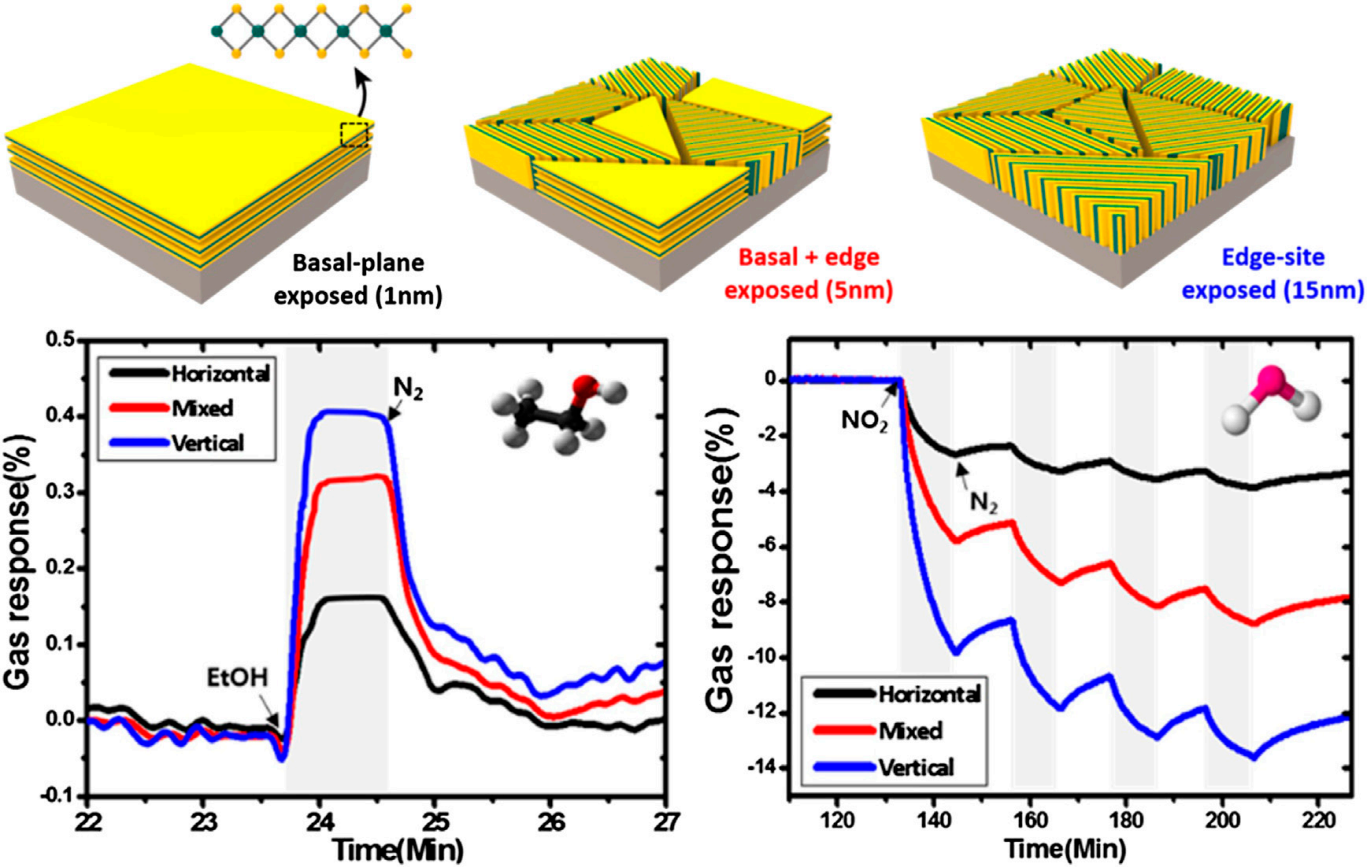
MoS_2_ edge sites (vertical) display higher sensitivity toward 0.1–100 ppm NO_2_ and 1,000 ppm ethanol in N_2_ compared to the predominant basal plane (horizontal) as a result of the greater number of active sites in the exposed edge site facets. Reprinted with permission from (Cho et al., 2015). Copyright 2015 American Chemical Society.

The performance and selectivity of 2D TMDC gas sensors may be improved through engineering the material surface and active sites of analyte interaction, adding trace dopants and photoexcitation. The first can be done by controlling the growth of the TMDC to expose a greater number of active sites on the surface. For example, *Cho et al.* increased the sensitivity of a MoS_2_-based room temperature sensor toward ethanol and NO_2_ by modifying the parameters of a CVD sulfurization process to induce different growth modes in the MoS_2_ (depicted in Figure 3), leading to vertical growth along a higher-energy plane with a greater number of active defect sites on the surface compared to a smaller number of active defect sites present on the horizontal basal plane (Cho et al., 2015).

**FIGURE 4:**
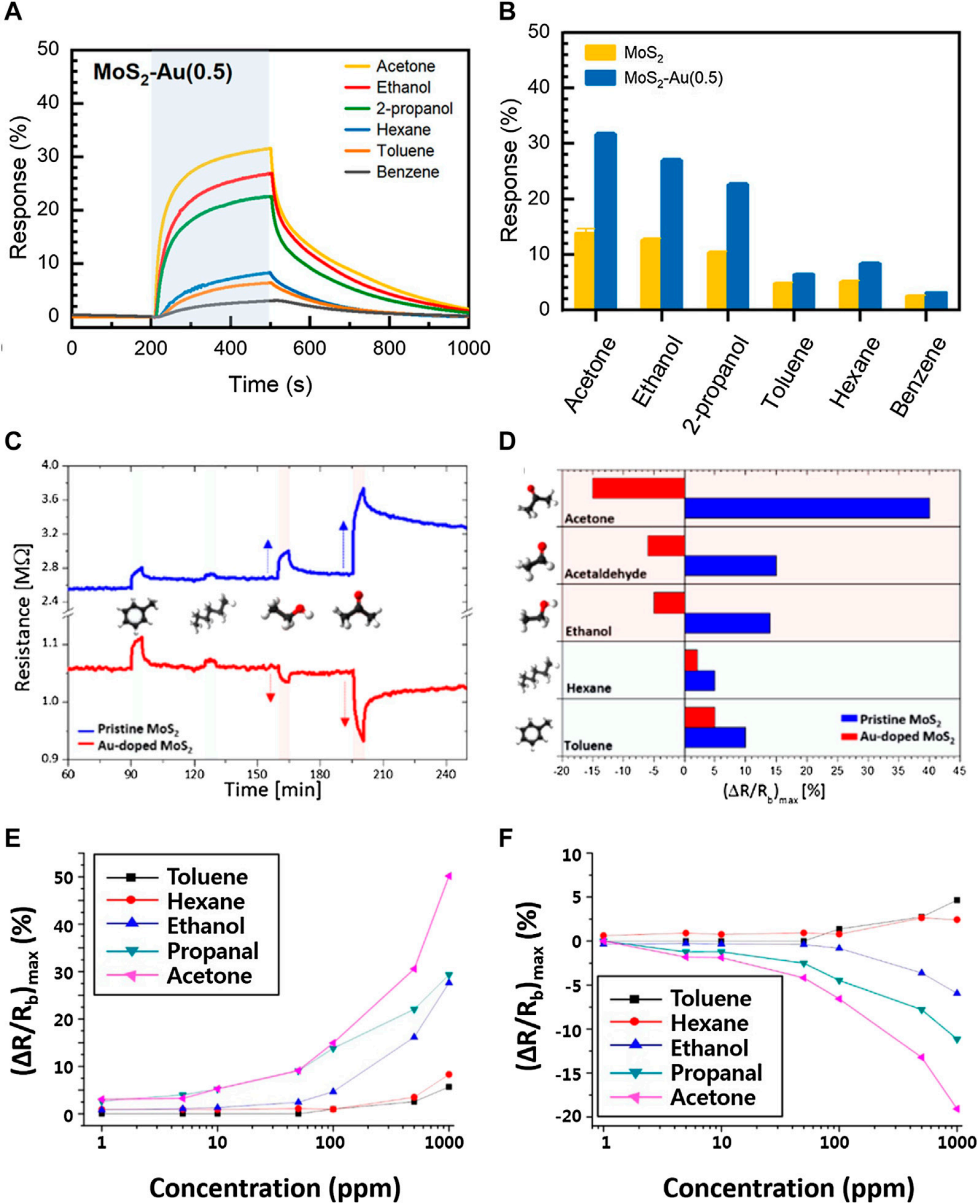
Dopants and surface functionalization modify the sensitivity and enhance the selectivity of 2D MoS_2_ toward different VOCs. **(A,B)** The sensitivity and selectivity of MoS_2_ toward oxygen-containing VOCs in air is increased when Au nanoparticles are embedded in the MoS_2_ nanosheets and exposed at the surface. Reprinted with permission from (Chen et al., 2019a). Copyright 2019 American Chemical Society. **(C,D)** The polarity of the response of MoS_2_ nanosheets toward oxygen-containing VOCs in nitrogen is reversed when Au nanoparticles are added to the nanosheets. Reprinted with permission from (Cho et al., 2017). Copyright 2017 American Chemical Society. **(E,F)** A similar reversal in response polarity toward oxygen-containing VOCs in nitrogen is also achieved when the MoS_2_ nanosheets are functionalized with mercaptoundecanoic acid ligands on their surface. Reprinted with permission from (Kim et al., 2014). Copyright 2014 American Chemical Society.

Differentiating between significantly different molecules such as NO_2_ and ethanol with pristine MoS_2_ nanosheets is clearly not difficult as seen in Figure 3, but differentiating between different VOCs, which is more applicable to some electronic nose applications, is more challenging with bare 2D TMDCs. One method of increasing the differentiability between VOCs using 2D TMDCs has been to add metal nanoparticles or organic ligands to the TMDCs. The functionalization of the MoS_2_ surface with Au nanoparticles (Cho et al., 2017; Chen et al., 2019a) and mercaptoundecanoic acid ligands (Kim et al., 2014) has been demonstrated to modify the sensing response to different VOCs, with both types of functionalized MoS_2_ nanosheets displaying higher increased selectivity and differentiability toward oxygen-containing VOCs vs. hydrocarbon VOCs—in one case even displaying a change in polarity of response when exposed to oxygen-containing VOCs (seen in Figures 4A,B)—when compared to bare MoS_2_. The carboxylic acid group in the mercaptoundecanoic acid was suspected to interact with the negatively charged oxygen atoms in the polar oxygen-containing VOCs via hydrogen bonding, leading to a charge transfer effect into the MoS_2_ nanosheets which affected their resistivity (Kim et al., 2014). On the other hand, different mechanisms for the sensitivity and selectivity enhancement toward oxygen-containing VOCs observed with the addition of Au nanoparticles were proposed: Au nanoparticles may act as a catalyst to increase the rate of or provide different pathways for the surface reactions with oxygen-containing VOCs (Chen et al., 2019a), while the p-type SiO_2_/Si substrate’s interface with the MoS_2_ nanosheets in combination with Au nanoparticles modified both the electronic structure and reactivity of the MoS_2_ compared to the pristine MoS_2_ (Cho et al., 2017). The experiments in (Cho et al., 2017) and (Kim et al., 2014) were carried out in nitrogen, while those in (Chen et al., 2019a) were carried out in air, leading to two different sets of sensing behaviors toward otherwise similar analytes; physisorption and dipole charge transfer alone are expected to be the dominant mechanisms in a nitrogen atmosphere, whereas adsorbed oxygen species potentially modify the mechanism in air by introducing a redox pathway. Within all three of these works, the Au nanoparticle and organic ligand-functionalization of the MoS_2_ nanosheets enabled greater differentiation between oxygen-containing and hydrocarbon analytes. Similar developments have also been observed with WS_2_ nanosheets, with Ag nanowires on the surface of WS_2_ nanosheets improving their selectivity toward NO_2_ over acetone and recovery performance by introducing NO_2_-selective catalytic active sites (Ko et al., 2016) and Au-nanoparticle functionalized WS_2_ nanosheets displaying higher selectivity toward CO over other VOCs due to the selectivity of active sites on the Au nanoparticles toward CO (Kim et al., 2020). This body of work shows that the functionalization of MoS_2_ and WS_2_ nanosheets with surface-exposed Au and Ag metal nanostructures and organic ligands is a viable strategy to differentiate between different VOC analytes and use in designing a cross-sensitive sensor array for an electronic nose. Of additional interest is the work of *Tuci et al.* (Tuci et al., 2018), which develops a method for the surface functionalization of MoS_2_ with a diverse class of organic ligands; this approach may be useful for fabricating a multifunctional TMDC sensor array with mixed organic surface ligands that display different affinities toward different VOCs.

Another technique for engineering the gas sensing performance of 2D TMDCs in electronic nose applications is through doping with transition metal ions. *Shao et al.* developed a cross-sensitive MoS_2_ nanosheet-based sensor array by varying the amount of Zn^2+^ incorporated into the nanosheets (Shao et al., 2018). A sensor array with MoS_2_ sensing elements containing different compositions of Zn^2+^ (ranging from 1 to 15% atomic) and operating at room temperature could differentiate between ozone (O_3_), N_2_O, formaldehyde (CH_2_O), ethanol and different mixtures thereof using a combination of kinetic and thermodynamic response features. Another application of transition metal ion doping included the substitutional doping of MoSe_2_ nanosheets with a small amount of Nb^2+^; this slightly decreased the nanosheets’ sensitivity to NO_2_ compared to the undoped MoSe_2_, but significantly increased the long-term stability of the baseline and gas sensing response by slowing oxidative degradation (Choi et al., 2017). *Panigrahi et al.* have provided future direction for the development of higher-performance 2D MoSe_2_ and MoTe_2_ gas sensing materials with computational predictions that doping them with low concentrations of Ge and Sb, respectively, will significantly enhance the sensitivity of these materials toward NH_3_, NO_2_ and NO (Panigrahi et al., 2019).

##### Conductive Polymers

Polymers are commonly used as insulators and are widely used in daily life due to their low-cost fabrication, optical and mechanical properties, thermal stability, and processability. The first polymeric sensors were obtained by dispersing conductive nanofillers into insulating polymers; however, the first conductive polymer was discovered by Green and Street in 1975 (Yi and Abidian, 2016). Conductive polymers are heterocyclic compounds with alternating single and double bonds along their backbone, creating conjugated π-electron systems (Rodriguez-Mendez and de Saja, 2020). Some commonly used conductive polymers include Polyaniline (PANI), Polypyrrole (PPy), Poly-3-methylthiophene (P3MT), Polythiophene (PT) (Wong et al., 2019).

These polymers can be arranged in various morphologies such as films (Joshi et al., 2011), thin films (Kumar et al., 2017), nanofibers (Yang and Li, 2010), nanowires (Zhang et al., 2009), nanowire arrays (Hassanzadeh et al., 2012), nanotubes (Yoon et al., 2006), nanoparticles (Lee et al., 2014), pellets (Chabukswar et al., 2001), nanoribbons (Lu et al., 2011), and other morphologies. The surface morphology of conductive polymers plays a significant role in the gas sensing performance, especially as nanostructures. Due to their larger specific surface area, 1D conductive polymers nanostructure are considered excellent material for gas sensing (Lu et al., 2011). It was observed that a thin layer of PPy sensor displayed a response of 1.38 in terms of the ratio of sensors resistance in air to its resistance in the presence of the target analyte to a concentration of 12,500 ppm of ammonia at room temperature (Chen et al., 2007), whereas a thin film of PPy displayed a response of 2.56 toward 10,000 ppm of ammonia at room temperature (Wong et al., 2019). Conductive Polymers are most commonly synthesized by chemical or electrochemical polymerizations of the corresponding monomers, but other methods can be used such as photochemical polymerization, metathesis, and plasma polymerization (Kus et al., 2018). The substrate and morphology have a significant influence on the sensitivity, reliability, accuracy, response time, susceptibility to interferences, and shelf life of the array. (Askim et al., 2013).

Conductive polymer-based sensors were used in the first commercial artificial nose system, and have demonstrated their effectiveness for vapor sensing (Yi and Abidian, 2016). Since then, polymers have been utilized in the development of a wide range of sensors due to their excellent transducing material and response to various chemical and physical stimuli (Guadarrama et al., 2000). The conjugated backbone consisting of alternating single and double bond structures displays electrical conductivity due to the sp^2^ hybridized carbon atoms on the polymer backbone. The parallel p orbitals above and below the carbon atom form a π bond constructs a pathway for the charge carriers transported along the polymer chains (Yi and Abidian, 2016). However, organic conjugated polymers generally lack intrinsic charge carriers. Therefore, external charge carriers are introduced to the polymers via doping. Conductive polymers can either be partially oxidized by electron acceptors or partially reduced by electron donors (Dai, 2004). Based on band theory, it is known that insulators have a large band gap between the conduction band and valence band. When conducting polymers are doped, either the valance or conduction band is filled, or polarons are generated during the process.

The conductive properties of the polymers are attributed to the intra and inter chain transports (Hangarter et al., 2013). In p-type sensing material, oxygen molecules from the air are chemisorbed onto the surface, removing electrons from the conduction band. The adsorbed oxygen is then converted into double or single oxygen ions, leading to ionosorption on the surface. The removal of electrons leads to a decrease in electron density and an increase in hole concentrations, which leads to a diminution in the resistance. As a reducing gas such as ammonia reacts with the ionosorbed double oxygen ion species, electrons are absorbed by the conduction band of the p-type material. As a result, the hole concentration decreases, and the resistance increases. When an oxidizing gas such as NO_2_ is introduced, electrons are depleted from the valence band, which leads to an increase in hole concentration and decrease in resistance (Arafat et al., 2012; Baharuddin et al., 2019). The sensing mechanism is the opposite for the n-type material. The sensing mechanism of carbon-polymer composites in artificial nose systems is observed in devices such as Cyrano Sciences’ Cyranose 320, which consists of 32 individual polymer sensors blended with carbon black composite (Dutta et al., 2002). The device is configured as an array of sensors, which swell when exposed to VOCs changing the conductivity of the carbon pathway. The swelling leads to a change in the resistance across the array, which is captured as a digital pattern that is a representation of the test smell. Each VOC regardless of its complexity has a distinct response signature and ‘smell print’, which is specific to the stimulus (Dutta et al., 2002).

Conductive polymers can be either n- or p-doped, and this doping provides charge carriers and modifies the band structure. Various counterions can be used as dopants to modify films to obtain various physicochemical properties. The dopants can also be deposited as thin films onto interdigitated electrodes using inkjet deposition, electrospinning, or electrodeposition to obtain films with various structures, hydrophobicity, thickness, and roughness (Rodriguez-Mendez and de Saja, 2020).

Due to their modifiable selectivity, short response time, ease of synthesis, mechanical properties, and capability to operate at room temperature, conductive polymers have been materials of interest for artificial nose application since the 1980s (Wilson and Baietto, 2009). Doped arrays of nano- and micro-structured conducting polymers doped with perchlorate (ClO^−4^), para-toluene sulfonate (pTS^−^), chloride (Cl^−^), Trichloroacetate (TCA^−^), dodecyl sulfonate (DS^−^), and dodecylbenzene sulfonate (DBS^−^) were capable of detecting acetone, methanol, ethanol, 1-propanol, 2-propanol, nitromethane, propylamine, pyridine, and gas mixtures of aliphatic alcohols (Alizadeh et al., 2016). Doping can also enhance the sensing mechanism of arrays of microchemiresistors. An electronic nose system was modified with bio-inspired nanofibrous artificial epithelium to produce a microchemiresistor covered with electrospun nano-fibrous structures that were prepared by blending doped poly emeraldine, with polyethylene oxide, polyvinylpyrrolidone, and polystyrene, which acted as the charge carriers for the conducting polymer (Alizadeh et al., 2016). Conductive polymer-based artificial nose systems have been utilized in the detection of bacterial wetwood detection in Fagus grandifolia and Prunus serotina Sapwood based on the detection of headspace volatile microbial and plant metabolites derived from sapwood. The sensor array of an Aromascan A32S conductive polymer along with principal component analysis and quality factor techniques could provide unique and identifiable aroma signature profiles for four healthy and wetwood-infected sapwood core types. The principal component analysis showed that not only were the healthy and wetwood-infected samples distinguishable, but there was a clear distinction between the profiles of the healthy American beech and black cherry sapwood cores (Wilson, 2014). In another example, conductive polymer-based E-nose systems were used for the discrimination of various olive oil samples based on their aromas. A system of eight different polymeric gas sensors were prepared by electrodeposition under varying conditions to produce and dope PPy, P3MT, and Polyacrylonitrile (PAN) thin films with different properties. The sensors were then repeatedly exposed to the headspace of olive oils and pattern recognition techniques were used to discriminate the signals. It was observed that each sensor had a unique response when exposed to the sample of olive oil. The response of P3MT sensors generated using different doping electrolytes to extra virgin olive oil is observed. This sheds light on the significant contribution of dopants to the sensitivity and selectivity of the generated sensors with respect to the different VOCs (Guadarrama et al., 2000). Furthermore, the response of the P3MT sensor array to various VOCs are observed. The sensor displays observable sensitivity to all the samples including the sample of flat olive oil. It is observed that various polymeric sensors prepared from different monomers and dopants displayed varying degrees of sensitivity when exposed to the headspace of an extra virgin olive oil. This is attributed to the different chemical natures of the monomers and the dopants used in the generation of the thin conductive polymer films.

### Optical Approach

Optical gas sensing is another useful mechanism in building an artificial nose; it can not only exhibit fast response with high selectivity and reactivity, but it can also be insensitive to electrical or magnetic noise. Depending on the optical properties of the sensing material such as refractive index, porosity, and optical transparency, optical gas sensors can provide unique response to the analytes.

In the context of optical detection modality, a photon is unique and valuable as it can generate specific patterns by monitoring not only the dynamic change of the number of photons transmitting through the sensing volume to identify the physical or chemically absorbed analytes, but also the wavelength, frequency (phase), and polarization of the photon motion at the same time. Intensity, measured by the number of photons, is the fundamental technique for the analysis of the photon motion. The change in the photon motion results from absorption, fluorescence, scattering, or refractive index change caused by the surrounding materials of the sensing volume. Absorption of UV or visible light by organic compounds is based on the transition of outer electrons (Baldini et al., 2006). Suppose the excited molecule by the absorption relaxes to its ground state through photon emission after non-radiation transition by vibration. In that case, fluorescence occurs, leading to an increased number of photons at a different wavelength. Scattering is another optical process that the light is scattered by analytes in random directions. Depending on the energy difference between the absorbed light and the emitted light, scattering can be classified as either elastic (Rayleigh) or inelastic (Raman) (Ferraro, 2003). While the total number of photons decreases if scattering occurs regardless of whether the scattering is elastic or inelastic, increase of the number of photons at redshifted wavelengths can be observed when Raman scattering occurs. Gaseous analytes can also change the refractive index of surrounding medium, resulting in the change in either the number of transmitted photons received by a photon detector or the phase difference with a reference optical mode (Hariharan, 2010). In practical applications, gas phase volatile molecules interact with light weakly because of its low concentration. As the result, relatively long interaction paths or large interaction volume is necessary to detect analytes in ppm concentration range, which then requires a large sensor volume and footprint (Goyal et al., 2017). It is prerequisite to enhance either the field intensity or surface area-to-volume ratio (S/V) to miniaturize the sensors. Nanostructured materials have demonstrated great potential in the field enhancement by confining the light within the nanostructured cavity, and have exhibited a high S/V. In this section, we cover three prominent optical sensing techniques based on cross-reactive nanoengineered materials which can create unique patterns in response to the multiple odorants.

#### Photonic Crystal (PhC)

PhC is a dielectric material with a periodic nanostructure that possesses photonic band structures. The periodic structure with a repeating high and low dielectric constant affects the propagation of electromagnetic wave within the structure. The reflection of the light within the periodic structure interferes constructively in accordance with Bragg’s law, as given by

$$m\lambda =2dn_{eff}sin\theta$$ (1)

where m is the diffraction order, λ is  the wavelength of the incident light, d is the lattice period of the crystal in the direction of propagation of light, $n_{eff}$ is the effective refractive index of the periodic structure, and θ  is the glancing angle between the incident light and the crystal plane (Yeh, 1988). A high reflection is observed when the wavelength of the incident light satisfies the Bragg condition, which depends on two factors: the lattice constant and the effective refractive index based on Eq. 1. The change of the $n_{eff}$ by the gas introduced to the space within the PhC results in the shift of the Bragg peak, providing the information about the molecules in the gas. The high specific surface area of the PhC allows a large amount of gas analytes to adsorb onto the surface, leading to the significant sensitivity to the change of the gas environment. Various nanostructured PhCs ranging from One- to three-dimensions have been fabricated for gas sensing (Goyal et al., 2017). The axis, which allows for modulating the reflection of the light, can be enhanced by increasing the PhC dimension. Due to the larger adsorption surface area and more modulating channels, the three-dimensional PhC chemical sensor has an advantage in multiplexing compared to those using the lower dimensional PhC. The change of the lattice distance as a detection route causes the significant variation in the reflected wavelength compared to that of the refractive index. Polymer-based PhC is one of the representative materials that offer the change of the lattice distance with the response to the interaction with the analytes. Control of the lattice distance can be considered as an additional modulating method to have analyte specificity but cannot be a main route due to the low sensitivity and response time compared to the refractive index. Porous silicon triggered the interest of porous structured (Vincent, 1994) as a PhC and it has been extended to the other materials for the last decades and utilized for various applications (Calvo et al., 2011; Lee et al., 2013). Highly matured Si technology enables the fabrication of highly periodically ordered structures and silicon has dominated the field of the porous PhC (Letant and Sailor, 2001; Gao et al., 2002; Lin et al., 2004; King et al., 2007b). It has been used for sensing various gases such as ethanol, acetone, hexane, etc. The vapor-phase species smeared into the pore of the PhC by capillary force changes the refractive index of the photonic crystal and the detection limit of the PhC varies depending on the refractive index of the analytes, ranging from ppb to pph.

**FIGURE 5:**
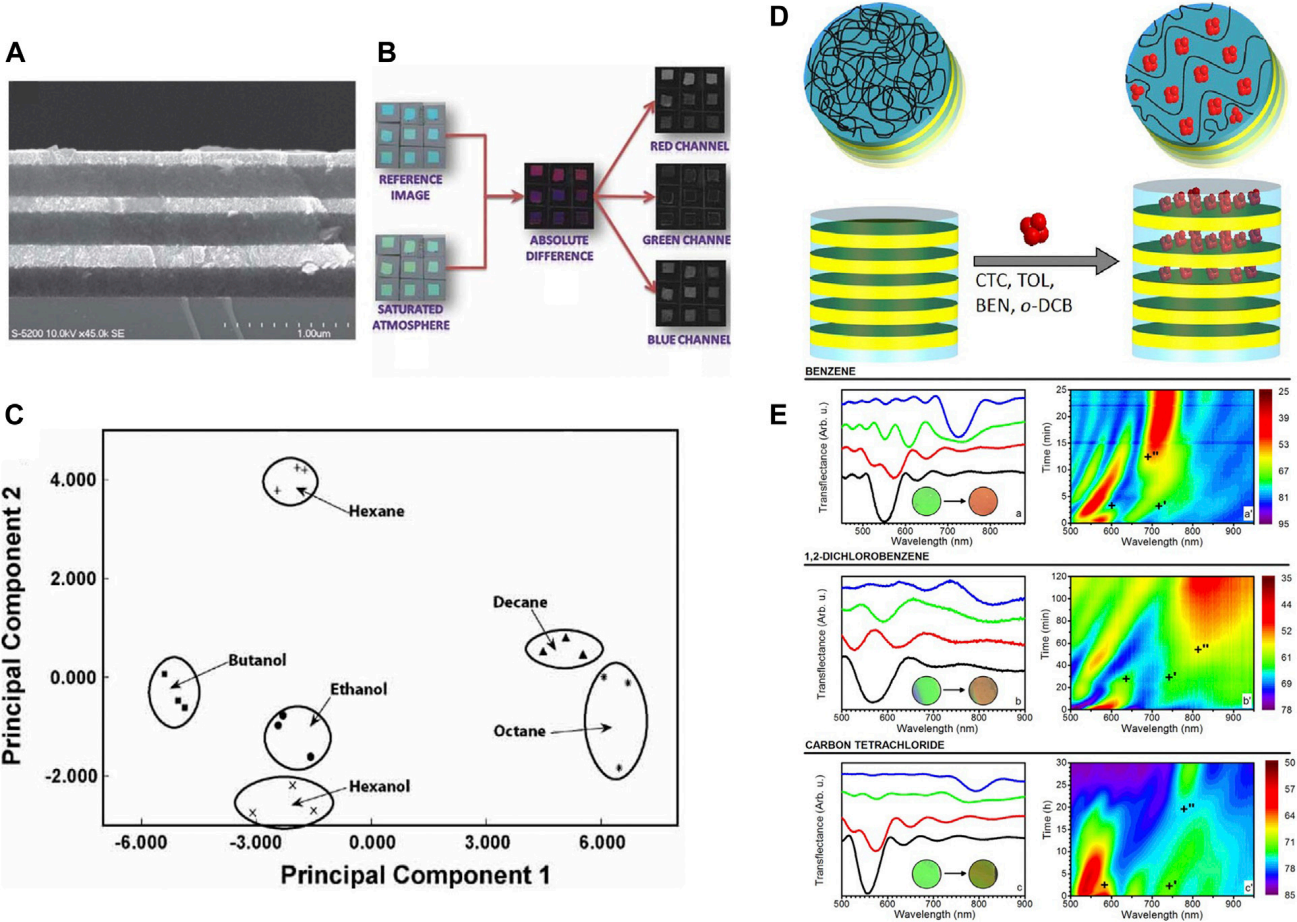
Photonic nose discriminating volatile organic compounds based on the effective refractive index **(A–C)** and the lattice constant **(D–E)**. **(A)** three bilayer Bragg stacks composed of SiO_2_ and TiO_2_ nanoparticle. **(B)** Color changes of the photonic-nose array upon its exposure to an analyte. **(C)** PCA plot of the color changes of the photonic-nose array. Reproduced with permission from (Bonifacio et al., 2010). Copyright 2010 Wiley-VCH-GmbH. **(D)** Schematic illustrating the change of the lattice constant through the formation of poly(p-phenylene oxide)/VOC cocrystals during the vapor exposure. **(E)** Time resolved optical response of the poly(p-phenylene oxide)-cellulose acetate upon its exposure to benzene, 1,2-dichlorobenzene, and carbon tetrachloride. Reprinted with permission from (Lova et al., 2016). Copyright 2016 American Chemical Society.

Many attempts to increase the sensitivity of the PhC have been conducted such as surface modification (Ruminski et al., 2008; Kelly et al., 2011) to increase the affinity of the analytes to the surface of the PhC and introduction of fluorescence dye (King et al., 2007a) to achieve analyte specificity. *Ozin et al*. have employed porous Bragg stacks (Figure 5A), which were comprised of (meso)porous multilayers and functionalized the surface of the pores with different alkoxysilanes that exhibited distinct surface energy characteristics (Bonifacio et al., 2010). The PhC pixels with different hydrophobicity could create unique color patterns for each gas and a successful discrimination between different alcohols and alkanes was demonstrated with 9 PhC pixels. Figure 5B represents the process in the color imagery analysis of the nine PhC pixels and the responses of each surface functionality for different analytes. 2D principal component analysis (PCA) plot (Figure 5C), which clusters the responses, allowed the assessment of the discrimination capabilities (Bonifacio et al., 2010).

PhC was also integrated with the materials that react with a specific gas resulting in the change of lattice constant of PhC, such as Pd for hydrogen (Lin et al., 2004), oxidized porous silicon for HF(Létant and Sailor, 2000), and SiH for Cl_2_ (Ruminski et al., 2011). For the multiplex analysis of the chemical species by utilizing the lattice constant variation, either different degree of analyte uptake by the PhC or formation of crystalline phases with a specific analyte is required. *Lova et al.* exploited the phase transition of Poly(p-phenylene oxide) from amorphous to cocrystalline phases induced by the absorption of VOCs (Figures 5D,E). The change of the crystalline phases upon the exposure to the different VOCs results in dissimilar optical properties, which can be detected even by naked eye (Lova et al., 2016). Despite of its label-free vapor selectivity, the detection configuration accompanying the crystal structure changes suffers from the low reversibility and chemical stability. The enhancement of the long-term stability must be addressed in the future studies for the configurations to be widely implemented.

##### Surface Plasmon

Surface plasmon based gas sensor was first demonstrated in 1983 (Liedberg et al., 1983) and remarkable growth has since been made over the last few decades as an analytical route in not only the gas detection field but also biology, energy, environment, and catalyst related fields (Le Ru and Etchegoin, 2008). Surface plasmons are the collective charge oscillations of free electrons in the metal caused by the interaction with electromagnetic waves. With the excitation of the surface plasmon, light is either coupled into propagating or standing surface plasmon depending on its structure. With the 1D or 2D plasmonic structure, propagating surface plasmon (PSP) can be excited and its propagation constant of the surface plasmon is given by the following expression:

$$\beta =k\sqrt{\frac{\varepsilon_{m}\varepsilon_{d}}{\varepsilon_{m}+\varepsilon_{d}}}$$ (2)

where k is the free space wave number, $\varepsilon_{m}\;and\;\varepsilon_{d}$ are the dielectric constants of the metal and environment, respectively. As the refractive index ($\varepsilon_{d}\;or\;n_{d})$ changes due to the gas molecules, the propagation constant changes, resulting in the change of the resonance condition between the surface plasmon and incident light. The change subsequently alters wavelength, frequency (phase), and/or polarization of the photon motion in the light in the output and allow for estimation of the refractive index of the adsorbed molecules on the metal. We refer reader to (Schasfoort, 2017) for more theoretical background of the surface plasmons.

**FIGURE 6:**
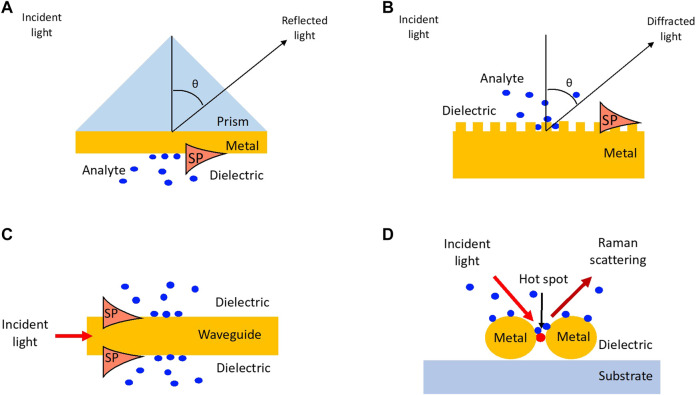
Surface plasmon based sensor configuration. **(A)** prism coupler-based PSP system, **(B)** waveguide-based PSP system, **(C)** grating coupler-based PSP system, **(D)** localized surface plasmon resonance (LSPR) or surface-enhanced Raman spectroscopy-based system.

**FIGURE 7:**
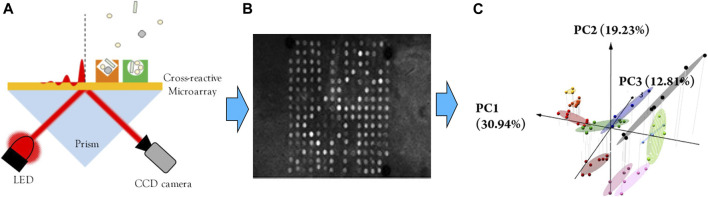
Highly selective optoelectronic nose based on surface plasmon resonance imaging. **(A)** schematic illustration of the setup of the optoelectronic nose. **(B)** SPRI differential image of the microarray recorded by the CCD camera after exposure to a VOC sample **(C)** PCA score plots for the discrimination of structurally similar VOCs. Reprinted with permission from (Brenet et al., 2018). Copyright 2018 American Chemical Society.

For the excitation of the surface plasmon, its momentum must match that of the incident light. The momentum of the surface plasmon is larger than the incident light in air based on the dispersion relation. The increase of the momentum can be fulfilled by prism, grating, and waveguide couplings, which are the common configurations for the excitation of the surface plasmon (Figure 6). For the prism coupling, the light wave in a high refractive index prism with a certain angle can generate an evanescent wave. If the momentum of the evanescent wave matches that of the surface plasmons, a PSP is excited at the other interface between the metal film and environment with gas. As the result, a significant reduction of the reflected light occurs due to the excitation of the surface plasmon at the angle. Another type of SPR excitation method is grating coupling as shown in Figure 6B. Here, the diffraction by grating is employed to increase the momentum of the incident light. The excitation of the PSP by prism and grating couplings depends on the optical properties of the dielectric and any change of the refractive index by the adsorption of the analytes on the metal surface subsequently alters the resonance angle of surface plasmon. The configurations are highly sensitive, simple, and reproducible, and therefore the technologies have been intensively utilized in the past 3 decades as a common sensing platform (Homola and Piliarik, 2006; Brenet et al., 2018). However, identification of gaseous components from multiple orders is still challenging by monitoring RI change alone. Multiple sensing arrays with different chemical specificity must be utilized to discriminate groups of target species. *Brenet et al*. have demonstrated the detection of VOCs with 72 cross-reactive sensor microarrays (Figure 7). The microarrays were functionalized with 18 biomimetic peptides and organic molecules by thiol-gold chemistry. Each peptide and organic molecule with different physicochemical properties (e.g., hydrophobic, hydrophilic, charged, neutral, etc.) allowed for distinguishing between homologous VOCs with the difference of a single carbon atom (Brenet et al., 2018).

Recently, nanoplasmonic sensors have attracted attention in the field of the chemical sensors due to their high potential for multiplex sensing and miniaturization of the plasmonic sensors. The nanoplasmonic sensors employ tightly localized surface plasmon (LSP) in the vicinity of the engineered metallic nanoparticles or nanostructures instead of the PSP on the metallic film for the conventional surface plasmon techniques (Figure 6D). The nanoplasmonic sensors can reduce the sensor volume and cost by utilizing a simple colinear transmission or reflection illumination system instead of bulky prism-coupling system for PSP techniques (Haes and Duyne, 2004; Gao et al., 2014). Chemical sensing with LSP exploits the two unique effects of the LSP: 1) electric fields in the vicinity of the plasmonic nanoparticles or in the small gap generated by the nanostructure are drastically enhanced and 2) the field enhancement has the maximum at the resonance frequency which depends on the size, gap distance, and chemical composition of the nanoengineered materials. The LSP resonance frequency or the wavelength, which are related with the refractive index of the environment, is as follows:

$$\omega_{max}=\frac{2\pi c}{\lambda }=\frac{\omega_{p}}{\sqrt{2\varepsilon_{d}+1}}$$ (3)

where $\omega_{p}$ is the plasma frequency, c is the light velocity in air, and λ is the wavelength of the light. PSP based gas sensor is known to have sensitivity to the bulk refractive index four times higher than that of the LSP sensor. (Haes and Duyne, 2004) The higher sensitivity of the PSP results from the large surface area and the long decay length (∼200 nm). With respect to the same footprint, however, the LSP can offer higher sensitivity (Bingham et al., 2010). By harnessing the tunable resonance frequency and well-developed functionalization chemistry of the plasmonic nanomaterials, a single nanoparticle could possess a potential to serve as an independent sensor taking the advantage of its high sensitivity and the short decay length (30 nm) (McFarland and Van Duyne, 2003; Svedendahl et al., 2009). In addition, the extremely large surface area of nanomaterials substantially provides enough sites for adsorption or condensation of the vapor, leading to the surface refractive index change. (Cheng et al., 2007) There have been numerous proof of concept studies demonstrating the discrimination ability of the LSP among different gaseous molecules (Karakouz et al., 2008; Chen and Lu, 2010; Joy et al., 2012); however, Au is the only material potentially applicable for the artificial nose due to its high chemical stability and high Q factor of absorption frequency (Le Ru and Etchegoin, 2008). Thus, its tunable wavelength range of surface plasmon with high sensitivity is limited at red or near IR wavelength, where resonance peak absorption is broadening due to the inhomogeneous size distribution of the large particles. Although the difficulty in the synthesis of uniform NPs and fabrication of highly ordered arrays prevented the sensor from being tuned at the most desired wavelength to reduce the absorption and scattering bandwidth (sharpness) of LSP, the ongoing advances in optical lithography technology will solve the issues with the band broadening of LSP and eventually enhance the discrimination capability between a multitude of odorants.

Analysis based on the vibrational spectroscopy has many advantages in that it can provide the fingerprint of the molecules directly, and therefore labeling the sensing materials with a chemical indicator or dense sensing arrays to create unique patterns corresponding to the multitude of analytes is not necessary. Among the various vibrational spectroscopy, Raman spectroscopy is the most widely used technique in chemical analysis, and it utilizes visible light, which does not often face interference from the main components of ambient air (nitrogen, oxygen and water vapor). But the Raman scattering is an extremely weak phenomenon; hence, detection of the dilute analytes such as gaseous odorants in atmosphere is not an easy task. LPSR can enhance the weak Raman scattering by up to 11 orders of magnitude, and it allows for vibrational fingerprint of even a single molecule to be observed (Le Ru and Etchegoin, 2008). This technique is called surface enhanced Raman spectroscopy (SERS), which can occur near the surface of metallic nanostructures, especially within a plasmonic hotspot, which is a gap smaller than a couple of nm created by metallic nanostructures. Due to the technical difficulty in uniformly fabricating such a small gap distance, six to eight orders of magnitude enhancement is realistic values with the good SERS substrates (Le Ru and Etchegoin, 2008). Compared to fluorescence or QD, the narrow bandwidth of the Raman scattering enables the integration of multiple adsorbents with the SERS arrays and the distinct peaks at different wavenumber can offer multiplex molecular target detection. Due to the extremely low Raman cross-section of the gas, however, detection of the analyte freely moving around in air is still not quite feasible. For this reason, adsorption of the gaseous molecules on SERS substrates is prerequisite. The adsorption of the gaseous molecules has been enhanced by either introducing the materials with a high affinity to the analytes (Rae and Khan, 2010), prolonging the retention time of the analytes to be adsorbed using dendritic Ag nanocrystals (Zhang et al., 2017), or applying electric field to the Ag-coated SiO_2_ beads to increase the adhesion of electrostatically charged molecules (Lin et al., 2013) with the gas sensitivity down to ppb level. Compared to other sensing techniques, the adsorption of the analytes without specificity is preferential for SERS-based sensor because the vibrational fingerprint of the molecules can be obtained directly. The high adsorption capacity for the enhanced SERS effects often causes the substrate contamination leading to the signal drift and loss of the function. Improvement of the reversibility is essential for continuous and effective operation over a long period of time.

#### Waveguide

**FIGURE 8:**
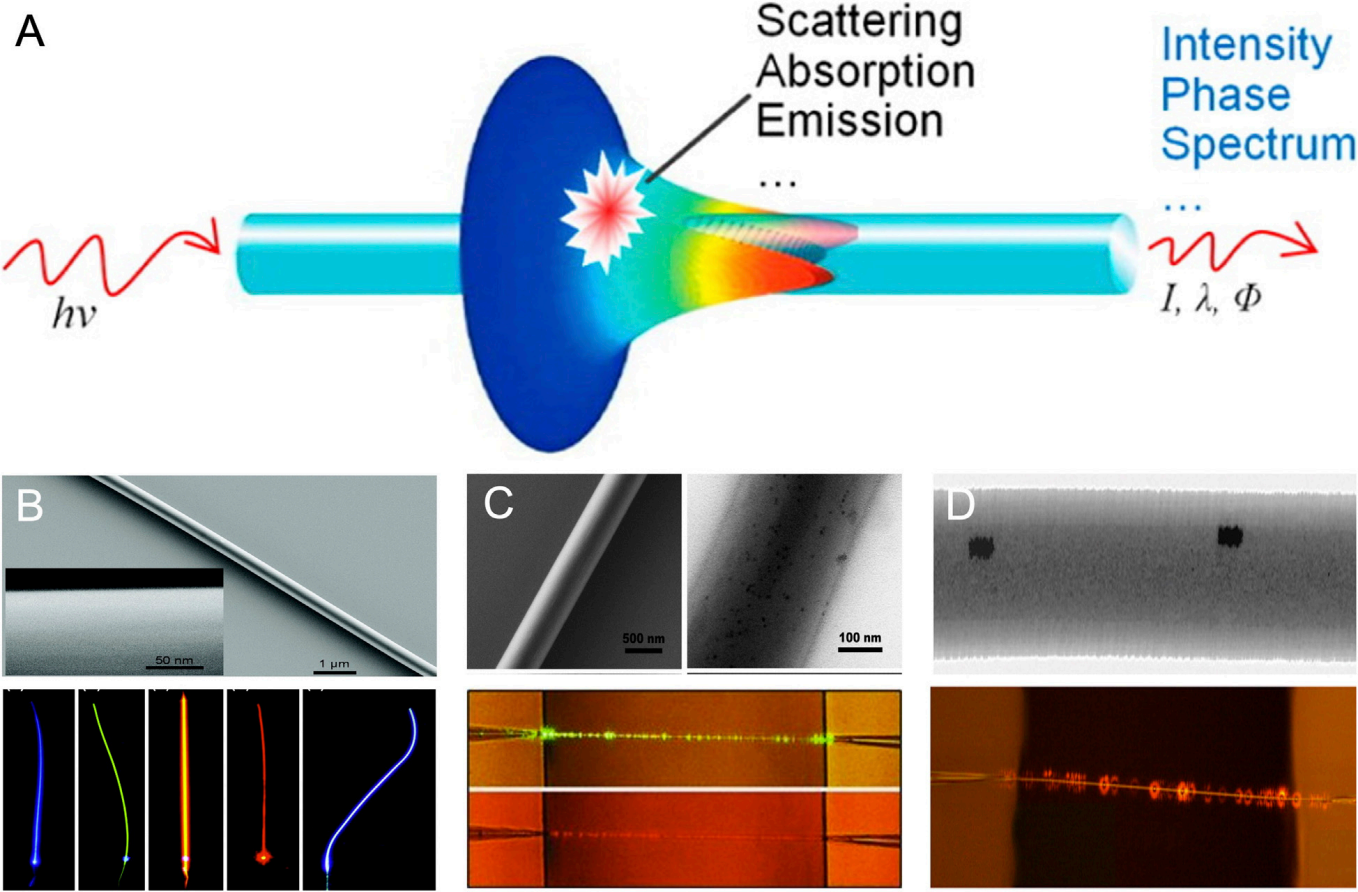
Nanoscale waveguide for optical sensing. **(A)** Schematic illustration of nanoscale waveguide with a high fraction of evanescence fields whose intensity, phase, and spectrum are vulnerable to the surrounding environment. Reproduced with permission from (LeiZhang, 2020). Copyright © 2020 Elsevier. Nano waveguide optical gas sensor doped with **(B)** fluorescence **(C)** quantum dot **(D)** gold nanoparticle. **(B)** Reprinted with permission from (Gu et al., 2010). Copyright 2010 American Chemical Society **(C)** Reproduced with permission from (Meng et al., 2011). Copyright Wiley-VCH GmbH. **(D)** Reprinted with permission from (Wang et al., 2012). Copyright 2012 American Chemical Society.

Nano- or sub micro-waveguide based chemical sensors provide a high-fractional evanescent field along the waveguide and allow for the guided light to be highly sensitive to the surrounding materials (Lou et al., 2005). It also offers small footprint, enabling creation of different patterns by increasing the number of different sensor arrays. In addition, the nanowire-based sensor arrays can be integrated with the nanowire lasers or detectors, which enables the versatile platform for fast-response, small foot print, and low power consuming optical sensing (Lin et al., 2013; Goyal et al., 2017). Owing to the well-established fabrication technique and high transmission in the visible and infrared wavelength region, fused silica glass fiber has been most popularly used (Jin et al., 2013). But, in the last decades, other materials such as polymer (Gu et al., 2008; Meng et al., 2011; Wang et al., 2012), silicon (Janeiro et al., 2019), semiconductor (Pauzauskie and Yang, 2006; Yan et al., 2009) or metallic nanowires (Kim et al., 2017) have emerged with their unique advantages for the guidance of the light within the nanoscale waveguide (Kim and Yan, 2018). Either refractive index change or absorption or emission of the analyte surrounding the waveguide will result in the change of the intensity, phase, polarization, or spectrum of the light in the output. Most of the chemical species have a strong absorption spectrum in both UV and infrared regions. The absorption in UV region of the spectrum is associated with the electronic transitions of the molecules while infrared regions are for vibrational or rotational transitions of the molecules (Hodgkinson and Tatam, 2012). The energy of UV light is such that it can excite all the gases near the sensing volume, leading to poor selectivity. In addition, the electronic transitions may also occur within the waveguides, resulting in the extinction of the transmitted light or photoluminescence. Therefore, IR has been commonly used for the absorption based gas sensors, but it has difficulties in multiplexing to form sensor arrays on the compact footprint due to the loss by the diffraction (Kim and Yan, 2018). Visible light has the most appropriate spectrum regions with regards to the guidance of the light within the nanoscale waveguide, but the absorption of gaseous molecules is extremely weak. Refractive index change or fluorescence have been employed as alternative detection techniques. For the refractive index-based sensor, the fiber interferometers are commonly used which split the light into two beams that propagate in different optical phases: one is exposed to the analytes and the other one is isolated from the environmental variation. The change of the optical path due to the refractive index change of the surrounding materials results in the change of the interference fringe (Li and Tong, 2008; Lee et al., 2012a; Chai et al., 2019). The refractive index change-based sensing platforms operate well in the environment that only one type of the known molecule exists with a high concentration such as liquid phase but have difficulties in distinguishing the multitude odorants due to the low refractive index contrast between the dilute gaseous molecules (Tong, 2018). Bragg gratings has been advent in recent years to improve the sensitivity of the sensor and could have reduced the fiber diameter and overall length (Liu et al., 2011). But the most demonstration of the sensitivity has been still limited to the detection of the refractive index of the liquid rather than gaseous environment (Liu et al., 2011; Xiao et al., 2016). Fluorescence dyes (Gu et al., 2008), 0- to 2-D nanostructures (Villatoro and Monzón-Hernández, 2005; Monzón-Hernández et al., 2010; Meng et al., 2011; Urrutia et al., 2015), and graphene layer (Bao et al., 2010; Wu et al., 2014; Yao et al., 2014a; Yao et al., 2014b; Irawati et al., 2017) have been integrated with the nanoscale waveguide sensor arrays to enhance the specificity to certain chemical species and to increase the sensitivity (Figure 8).

Fluorescence based chemical sensors provide outstanding sensitivity and have flexibility for choice of the spectra region. Strong emission spectra of the fluorescence at longer wavelengths than its absorption spectra allow for easy filtration of the incident light (Baldini et al., 2006; Lakowicz, 2013). Despite these advantages, the fluorescence suffers from photobleaching, which is the main bottleneck to their use in the artificial nose with a long-term operation stability.

Compared to organic dyes, quantum dot (QD) has a long-term photochemical stability, relatively high quantum yield, broad absorption spectrum and narrow emission band of almost symmetric shape. Flexibility of emission spectral position by particle size control is another attractive property of the QD for multiplexing (Resch-Genger et al., 2008). While functional groups of fluorescence dyes bind to a specific molecule, the QD can bind to multiple odorants in the similar manner with the receptors in olfactory system. *Meng et al*. have synthesized a CdSe/ZnS QD-doped polystyrene nanofiber and successfully demonstrated humidity detection with the QD-activated nanofiber with a fast response (90 ms) and low power consumption (Meng et al., 2011). Typical materials of the QDs are chalcogenide semiconductors made with group VI: CdTe, CdSe, ZnSe, and ZnS. Even though QD has potential as a route for the realization of artificial nose, the low reproducible physicochemical properties of the QD limits the multiplex chemical species detection (Resch-Genger et al., 2008).

Direct change in the transmission by scattering and/or absorption of the incident light in response to the environment is another strategy for the identification of surrounding analytes, and its sensitivity and selectivity can be enhanced by integrating the waveguide with additives such as palladium nanoparticles (Sirbuly et al., 2008) or nanofilm (Villatoro and Monzón-Hernández, 2005), Au nanorods (Wang et al., 2012), or graphene (Bao et al., 2010).

While the light travels in dielectric waveguide by total internal reflection being confined within the waveguide, one in the metal travels along the interface between metal and dielectric enabling the guidance of the light beyond the diffraction limit. Accordingly, more unique response pattern for the multitude analytes can be created by packing more dense sensing arrays on a limited footprint. Enhanced interaction between light and analytes due to the tightly confined evanescent fields traveling on the surface of the metal waveguide offers the significant improvement of the sensitivity to the change of the refractive index of surrounding (Figure 6C). (Ordal et al., 1983; Homola and Piliarik, 2006; Wei et al., 2013) Despite these unique advantages, the detection of gas using the plasmonic nanowire waveguide has not been reported yet possibly due to the significantly large propagation loss from the ohmic damping, leaky plasmonic modes, and scattering (Lopez et al., 2017). There have been many attempts to reduce the propagation loss by using a chemically synthesized metallic nanowire with an ultra-surface smoothness (Kim et al., 2017), hybrid waveguide (Oulton et al., 2008), and gain materials to compensate the loss (Oulton et al., 2009). However, further improvement may be required to be able to discriminate the extremely small change of the refractive index by gas.

Compared to the photonic crystal and surface plasmons, the artificial nose with cross-reactive nanoscale waveguide arrays is still at an infant stage. To be admitted as a leading candidate for the artificial nose, further development of auxiliary components such as the interconnector, coupler, modulator, etc. must be made (Kim and Yan, 2018).

### Optoelectrical Approach

For the last decades, the electrical and optical approaches have been considered as a prime candidate for the artificial nose and a great deal of research has been conducted to demonstrate the superiority of each approach. In recent years, however, there have been new attempts to combine both electrical and optical approaches to overcome their individual drawbacks and/or to create more unique response patterns for the multitude of odorants providing more information about the molecules’ identity. This new strategy monitors the motion of the electron or photon under the variation of the other transducer or both together in response to the environmental change.

#### Optically Modulated Electrical Sensor

Light has been utilized for the photoexcitation of the semiconductor in which electrons are excited from the valence band to the conduction band, increasing the surface density of electron charge carriers. In metal oxides, these additional electrons are able to react with oxygen to create a larger surface concentration of reactive surface-bound oxide species which are then able to react with analytes (Xu and Ho, 2017; Kumar et al., 2020b). Since Tatsuma’s group found charge transfer from Au or Ag nanoparticles into TiO_2_ sol-gel films (Tian and Tatsuma, 2004), metallic nanoparticles also have been integrated with the semiconductor, allowing the excitation wavelength from UV to near infrared to be controlled by size of the metallic nanoparticles. The local temperature increase and hot electron generation by LSPR offer significant enhancement of the photocatalytic activity and enable the catalytic reactions to occur at room temperature (Tian and Tatsuma, 2004; Linic et al., 2011). Because the mechanisms of chemical sensing and catalysis are both considered to be electrochemical processes occurring between the metal oxides and gas molecules, this optical-enhancement strategy was applied to achieve room-temperature gas sensing for MOS-based sensors. In 2013, *Wang et al.* demonstrated enhanced gas sensing performance at room temperature with gold nanoparticle embedded into wide band gap silica nanowire (SiOx NW) (Wang et al., 2013). Many other different metal oxides with AuNPs have been used for room-temperature for the detection of a specific gas (Gogurla et al., 2014; Xu et al., 2018a; Zhang et al., 2018).

Although there have not been any reports demonstrating the pattern creation for the multiple odorants with light-activated metal oxide sensors, it is worth noting that photoexcitation can enhance the absorption of specific molecules (Juan et al., 2011; Barik et al., 2014; Galvan et al., 2018). Additionally, photoexcitation can modulate the chemical reaction pathways by selective excitation of LSPR with the different resonance wavelengths depending on the size and composition of the plasmonic nanoparticles. It can offer more information with a single sensing array about the chemical compositions compared to just a metal oxide-based chemiresistive sensor.

The strategy of light-enhanced gas sensing has been extended to the modulation of its selectivity to a specific analyte. TMDCs are another material for developing an electronic nose sensor array with tunable gas sensing properties. Some semiconducting TMDCs are photochemically active in the UV, visible and/or IR regions. This has been exploited to engineer TMDC-based gas sensors whose gas sensing performance changes when exposed to certain wavelengths and intensities of light. Illumination can modify or enhance the sensitivity, selectivity, and response/recovery times of 2D TMDC materials.

**FIGURE 9:**
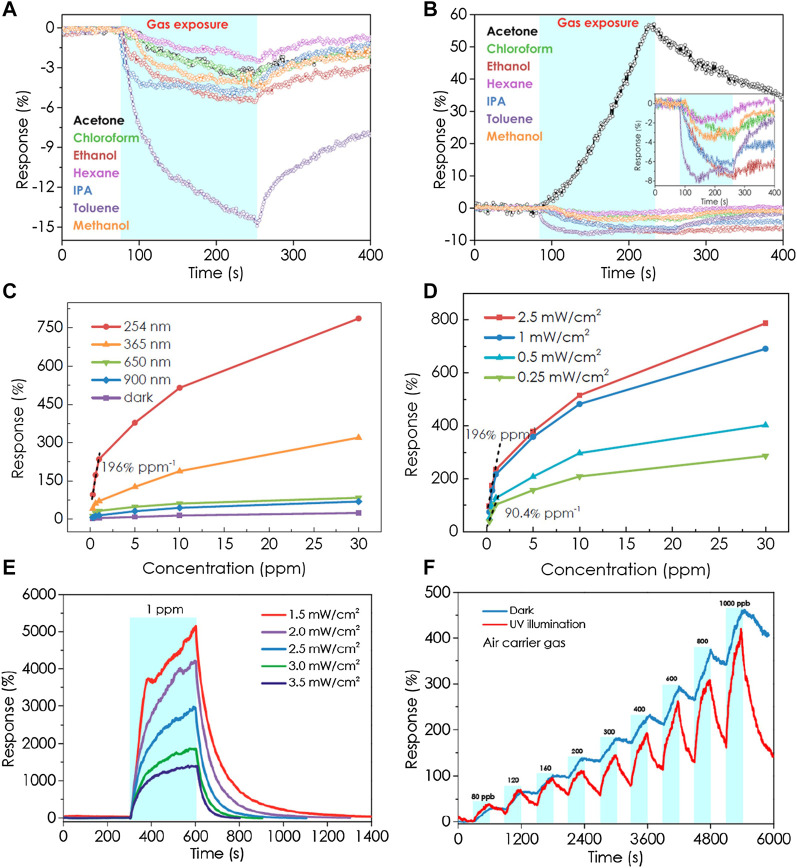
Light-enhanced VOC sensing of MoTe_2_. **(A)** shows the response of an MoTe_2_ FET sensor toward several VOCs at 100 ppm with no light illumination, while **(B)** shows the responses when the sensor was illuminated with 254 nm UV light. Reprinted with permission from (Wu et al., 2018a). Copyright 2018 American Chemical Society. **(C)** shows the dependence between response, concentration, and the wavelength of light illumination for an MoTe_2_ sensor exposed to NH_3_, and **(D)** shows the dependence between response, concentration and illumination intensity at 254 nm for the same sensor and analyte. Reproduced from (Feng et al., 2017b). **(E)** shows the response of an MoTe_2_ sensor exposed to NO_2_ and 254 nm UV illumination at different light intensities, and **(F)** shows the response transients toward NO_2_ under no illumination and 254 nm UV illumination. Reprinted with permission from (Wu et al., 2018b). Copyright 2017 American Chemical Society.

Some research in this area has specifically focused on UV light-excited MoTe_2_ FETs. MoTe_2_ under 254 nm UV illuminated displayed enhanced sensitivity and lower limits of detection toward NO_2_ and NH_3_ (Figure 9). (Feng et al., 2017b; Wu et al., 2018b) Illumination at this same 254 nm wavelength was also demonstrated to significantly enhance selectivity and reversed polarity toward acetone over humidity and other VOCs, including larger ketones which had the same reversed polarity as acetone but much lower sensitivities, compared to no illumination (Wu et al., 2018a). Since these experiments were carried out in air, it was suggested that the UV light promotes the desorption of oxygen from Te vacancies on the MoTe_2_ surface, leading to the enhanced sensing performance toward NH_3_ and NO_2_ due to the greater number of active sites available for interacting with the analytes, while acetone underwent a photochemical reaction to yield a reactive oxidizing species that strongly interacted with the MoTe_2_ surface.

Similarly, the selectivity toward NH_3_ of WS_2_ compared to other VOCs was enhanced by illumination at both infrared (940 nm) and UV (365 nm) wavelengths (Gu et al., 2018). Different mechanisms were proposed for the enhanced selectivity at the different wavelengths; the infrared light closely matched the bandgap of WS_2_, generating more excitons and a greater number of adsorbed oxygen species at the surface while modifying the pathway of NH_3_ reacting with adsorbed oxygen at the surface, while the UV light possibly generated an excited state of NH_3_ which had better orbital overlap with and more effective charge transfer toward the WS_2_ surface.

**FIGURE 10:**
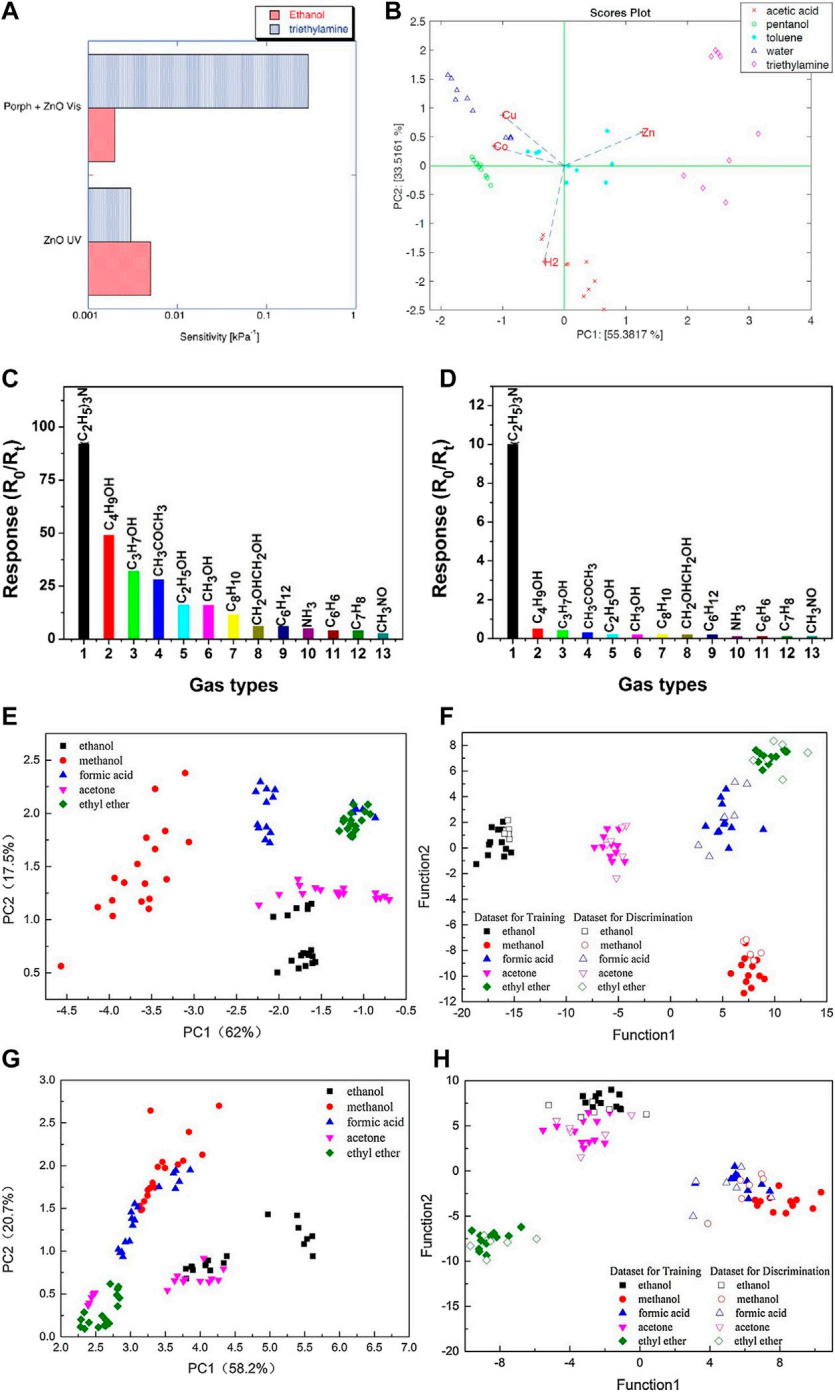
Light-modulated responses and selectivity in metal oxide gas sensors. **(A)** porphyrin-coated ZnO displays greater selectivity toward triethylamine over ethanol when excited with visible light, whereas pristine ZnO is more sensitive to ethanol under UV illumination. Reprinted with permission from (Sivalingam et al., 2012) Copyright 2012 American Chemical Society **(B)** The principle components extracted from the gas sensing results of photoexcited porphyrin-coated ZnO nanostructures with variable metal centers (indicated by the red text) (Magna et al., 2017). **(C)** The responses toward various VOCs of a ZnO/ZnFe_2_O_4_ composite at 240°C and with no illumination vs. **(D)** the responses at 80°C and with light illumination (wavelength >320 nm). Reprinted from Sensors and Actuators B: Chemical, 236, Liu et. al, Temperature & light modulation to enhance the selectivity of Pt-modified zinc oxide gas sensor, 350 - 357, Copyright 2016, with permission from Elsevier. (Liu et al., 2016). **(E,F)** The PCA (principal component analysis) and FDA (Fischer Discriminant Analysis), respectively, of features extracted from the responses of Pt-doped ZnO toward various VOCs using temperature and light modulation only. Reprinted from Sensors and Actuators B: Chemical, 247, Deng et. al, Temperature & light modulation to enhance the selectivity of Pt-modified zinc oxide gas sensor, 903 - 915, Copyright 2017, with permission from Elsevier. (Deng et al., 2017). **(G,H)** The PCA and FDA, respectively, of features extracted from the responses of Pt-doped ZnO toward various VOCs using temperature modulation only. Reprinted from Sensors and Actuators B: Chemical, 247, Deng et. al, Temperature & light modulation to enhance the selectivity of Pt-modified zinc oxide gas sensor, 903 - 915, Copyright 2017, with permission from Elsevier. (Deng et al., 2017).

The coating of metal oxide nanostructures with aromatic organic compounds has also been used as a strategy to impart sensing modulation based on the presence or absence of visible light. Porphyrins are a diverse class of heterocycle which may contain a coordinated metal center and various functional groups covalently bound to a central porphine ring. Pyrenes consist of four central and conjoined aromatic rings with covalently bound peripheral functional groups. Both the peripheral functional groups and, for porphyrins, the metal center, can be varied, which has allowed for the fabrication of ZnO-based sensor arrays whose individual sensors have varied sensing properties based on the specific porphyrin or pyrene coating. Furthermore, the conjugation of porphyrins and pyrenes leads to their absorption in the visible spectrum, allowing for their gas sensing properties to be modified by the presence or absence of visible light. The development of such light-modulated sensors and sensor arrays based on porphyrin-coated (Sivalingam et al., 2012; Sivalingam et al., 2013; Magna et al., 2014; Mosciano et al., 2015; Magna et al., 2017) (Figure 10A) and pyrene-coated (Sivalingam et al., 2016) ZnO nanostructures have been previously reported. The variation of the metal centers in the porphyrins allowed for additional modification of the sensitivities of the porphyrin-coated ZnO sensors and was sufficient to allow the analytes, but not their concentrations, to be differentiated (Figure 10B). (Magna et al., 2017)

Similarly, both light and temperature modulation were combined to accurately distinguish between ethanol, methanol, formic acid, diethyl ether and acetone using Pt-modified ZnO nanoparticles (Deng et al., 2017). The addition of light modulation increased the analyte classification accuracy to 100% (Figures 10E, 10F), compared to the 95.55% classification accuracy that was achieved from temperature modulation alone (Figures 10G, 10H). Additionally, the selectivity of a ZnO/ZnFe_2_O_4_ composite toward triethylamine over several other VOCs was improved by reducing the operating temperature and irradiating the sensing material with a xenon-mercury light source at wavelengths greater than 320 nm (Figures 10D, 10C). (Liu et al., 2016)

#### Electrically Modulated Optical Sensor

**FIGURE 11:**
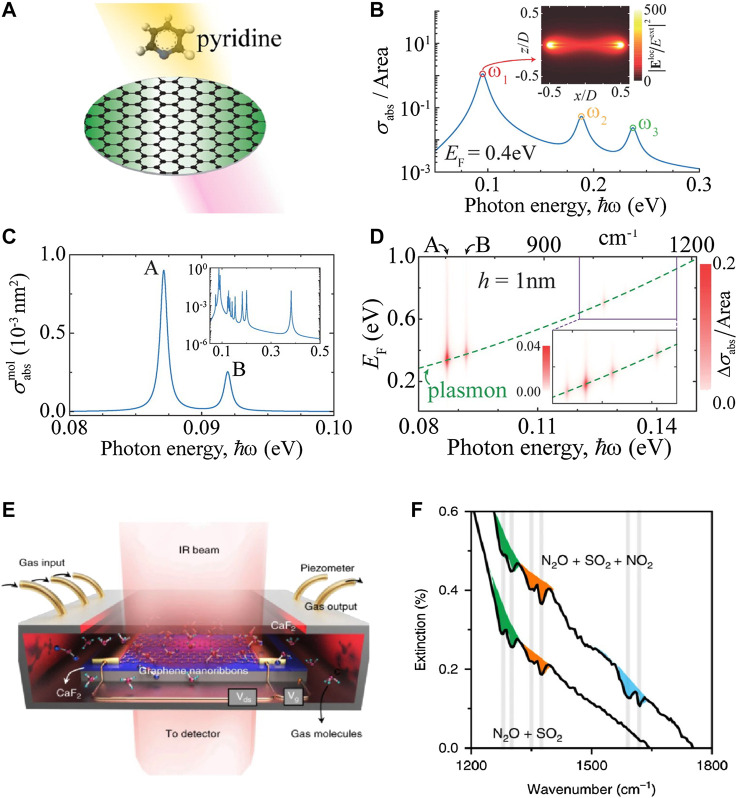
Surface-enhanced infrared absorption (SEIRA) spectroscopy with graphene plasmons. **(A–D)** SEIRA of pyridine molecule adsorbed onto the surface of graphene nanodisk. **(A)** Schematic of pyridine molecule placed 1 nm distance from the graphene disk **(B)** Absorption cross section spectrum of the graphene nanodisk normalized to its area at the Fermi level of 0.4 eV. **(C)** Absorption cross section spectrum of pyridine molecule. **(D)** Change in the absorption cross-section owing to the adsorption of the single pyridine molecule onto the disk plotted as a function of photon and graphene Fermi energies Adapted with permission from (Marini et al., 2015). Copyright 2015 American Chemical Society. **(E–F)** Identification of different nitrogen oxides by graphene plasmon based SEIRA. **(E)** Experimental schematic illustration of the graphene plasmon based SEIRA. **(F)** Absorption spectra of graphene in the presence of two gas mixtures: 1) SO_2_ and N_2_O 2)SO_2_, N_2_O, and NO_2_. (Hu et al., 2019a).

In optically enhanced metal oxide sensors, electronic properties of the sensing materials are modulated via optical stimulation. Conversely, an optical sensor modulated by electrical stimulation is another sensing modality for the optoelectrical approach as described in the following examples. Owing to its remarkable carrier-density tunability (Das et al., 2008; Schwierz, 2010) and surface sensitivity (Schedin et al., 2007), graphene has been integrated with electrical sensors to increase the sensitivity to the change of the environment for the last decades. Recent studies have focused more on the optical properties of graphene, such as the SPR characteristics of graphene in the visible to near-infrared wavelength range (Garcia de Abajo, 2014) due to its high modal field confinement and field enhancement which enable strong light-matter interactions (Yao et al., 2014a; Hu et al., 2019a). Furthermore, the dynamic tunability of graphene SPR characteristics by varying the electrostatic gate voltage has been demonstrated to influence the molecular interactions between graphene and gas molecules (Magna et al., 2014), offering additional identity information about the adsorbed gas (Kulkarni et al., 2016) (Figures 11A–D). On top of this, Fermi-level of graphene is one-to-one correlated with its plasmon resonance where a strong absorption of the light occurs (Figure 11D). Therefore, the spectral information of the molecules adsorbed to graphene can be resolved by examining the change of the broadband light absorption as a function of the Fermi level rather than the photon energy. Consequently, costly and bulky laser sources and spectrometers are no longer necessary for identification of the spectral signatures of the molecules. Following the similar strategy, the Raman scattering emission can also be enhanced by graphene plasmon and their spectrum can be analyzed as function of the Fermi level (Marini et al., 2015). After the demonstration of this new great strategy by numerical calculation, *Hu et al*. has experimentally measured the absorption spectra of gas molecules using the graphene plasmon based SEIRA and distinguish between the gas molecules which have similar compositions (Figures 11E, 11F). The study showed a great potential of the graphene plasmons for the label-free chemical identification without spectrometer and laser source which are the major bottlenecks in the development of cost-effective vibrational spectroscopy based artificial nose (Hu et al., 2019a).

## Discussion and Prospective

In this paper, we have generally overviewed both electrically and optically transduced gas sensors and their approaches to create unique response patterns for the multitude of odorants. Compared to the other sensing techniques, the electrical and optical sensing techniques integrated with nanoengineered materials allows for creating more dense sensing arrays on a limited footprint for discriminating multitude analytes.

Due to its simplicity, portability, and compatibility with standard electronics, the electrical approach, especially metal oxide-based sensors, has ruled the artificial nose for the last few decades. However, its high operating temperatures and sensitivity to humidity, and the limited information about the analytes carried by the electron motion give room for entry of optically transduced sensor with the potentials: 1) it can operate at room temperature and 2) it has less sensitive to humidity and 3) the light-matter interaction can provide multiple complex information. But spectroscopy, laser, and other optical components are expensive, and their size is still bulkier than the electrical sensor, which ends up imposing a constraint on mobility.

In the same manner in which multiple analytical techniques can be used to increase the reliability of identification and characterization of an unknown material, multimodal electrical and optical sensing can provide complementary information about the odorants’ identity. Recently, the two approaches have been combined and have demonstrated an enhanced sensing performance compared to using one sensing modality. The combination of the two transduction methods enables generation of more diverse response patterns toward odorants and enhancements of other sensing performance characteristics such as sensitivity, compactness, cost, etc.

The artificial nose with both optical and electrical sensing techniques is still in its infancy. However, there is still potential for the improvement of the artificial nose performance with precise structural and compositional control of the nanoengineered materials, which allow for increase in diversity of sensing materials, which would further generate more unique response patterns. Furthermore, combining transduction mechanisms of electron-matter or photon-matter interaction into an optoelectrical sensor array provides another possible set of response features.
